# Carbon reduction potential and selection strategies of emerging construction-phase technologies

**DOI:** 10.1038/s41598-026-39122-1

**Published:** 2026-02-09

**Authors:** Zhiping Liu, Zongjun Xia, Jing Li, Yifei Wang, Yufeng Zhang, Xiaodan Li, Qi Yang

**Affiliations:** 1 State Key Laboratory for Tunnel Engineering, China University of Mining and Technology (Beijing), D11, Xueyuan Road, Haidian District, Beijing, 100083 China; 2 School of Mechanics and Civil Engineering, China University of Mining and Technology (Beijing), Beijing, 100083 China; 3 China Railway 15th Bureau Group Co., Ltd., Shanghai, 200070 China; 4 The City Construction Company Ltd of China Railway 15Th Bureau Group, Luoyang, 471000 China

**Keywords:** Carbon reduction capacity, Carbon reduction pathway, Construction phase, Emerging technologies, Technology selection, Energy science and technology, Engineering, Environmental sciences

## Abstract

Carbon reduction has emerged as a critical global challenge. The building sector, as the primary contributor to carbon emissions, stands to benefit substantially from the adoption of emerging technologies during construction. This study quantitatively evaluates the carbon reduction potential of 25 emerging construction technologies, investigates their reduction pathways, and provides a comparative analysis and classification of these techniques. The results demonstrate that renewable resources and waste reduction technologies offer substantial reductions in carbon emissions from energy consumption and waste transportation, presenting a key avenue for achieving carbon neutrality in the construction phase. Prefabrication technologies, which relocate traditional on-site curing processes to factories while retaining only assembly tasks on-site, reduce emissions by over 90%. Technologies streamlining construction processes exhibit varying reduction rates, influenced by their impact on workflows. High-strength and high-performance materials, which optimize concrete and steel use on-site, show the least reduction (< 30%). A phase-specific technology selection strategy is proposed and validated through a case study, which demonstrates a 28.49% reduction in on-site emissions during construction through the integrated application of multiple technologies. In conclusion, this study quantifies the carbon reduction potential and pathways of emerging construction technologies, offering data-driven insights for technology selection, industry adoption, and policy development.

**Supplementary Information:**

The online version contains supplementary material available at 10.1038/s41598-026-39122-1.

## Introduction

Climate change and global warming are increasingly urgent global challenges^1^. Following the Paris Agreement’s goal of limiting warming to below 2 °C, many nations are actively working to reduce carbon emissions across all sectors. The building industry, a critical sector of economic development, plays a significant role in this effort. However, from material production and transportation to construction, operation, maintenance, and demolition, buildings contribute heavily to greenhouse gas emissions and global warming. According to the United Nations Environment Programme, the building sector accounts for 40% of global energy use and 38% of greenhouse gas emissions^2^, making it a major contributor to carbon output^3^.

Energy consumption and carbon emissions are present throughout the entire lifecycle (LCA) of a building^4^. Operational carbon (70%) and embodied carbon (30%) represent the two primary components of carbon emissions in a building’s lifecycle. Kang et al.^5^ defined operational carbon as CO_2_ emissions resulting from building operations, such as heating, cooling, and lighting, while embodied carbon refers to emissions linked to material production, transportation, construction, and demolition processes. Pomponi et al.^6^ noted that most researchers focus on reducing operational carbon through energy-saving strategies, yet Crawford^7^ underscored the significant role of embodied carbon in lifecycle emissions. Recent studies on embodied carbon have predominantly focused on material-related emissions^8^ (e.g., concrete^9^ and steel^10^) and emissions from prefabricated assembly processes^11^ (e.g., rebar cages^12^ and modular walls^13^).

Although construction-phase emissions represent a smaller proportion of a building’s embodied carbon, their impact is significant due to their concentrated and intense nature. In contrast to operational emissions, which are spread over 70 years, construction-phase emissions typically occur within 1–2 years. Advances in materials, energy systems, and equipment have facilitated the widespread adoption of emerging construction technologies. Jafarynasab et al.^14^ analyzed a high-rise building in Tehran, using material, transportation, and energy input data, and found that waste transportation accounted for 14% of construction-phase emissions—a reducible portion through waste minimization technologies. Similarly, Tabrizikahou et al.^15^ demonstrated that advanced waste reduction techniques for wood, concrete, and metal residues further mitigate emissions. Padilla-Rivera et al.^16^ quantified the carbon footprint of timber-framed residential buildings in Quebec, Canada, and found that prefabrication technologies reduce construction-phase emissions compared to conventional methods. Monahan et al.^17^ compared traditional and emerging construction techniques across two buildings and reported a 34% reduction in emissions with the use of emerging technologies. Renewable energy applications, which reduce fossil fuel and electricity consumption, also show considerable emission reduction potential^18^. These studies collectively confirm the carbon reduction capabilities of emerging construction-phase technologies. While existing research predominantly focuses on individual emerging technologies, comparative studies across multiple technologies remain scarce. This gap results in a lack of clear classification regarding the characteristics and applicable contexts of new technologies, hindering the development of informed technology selection strategies for the construction phase.

This study addresses this gap by systematically evaluating the carbon reduction potential of 25 emerging construction technologies. By calculating carbon emission coefficients and analyzing reduction pathways, the study compares and categorizes these technologies to clarify their characteristics and applicable contexts, thus facilitating technology selection. Additionally, a case study of a building project in Xinyang, China, quantifies emissions reductions achieved through the integrated application of multiple technologies, providing empirical evidence to support industry-wide adoption and inform policy decisions.

## Materials and methods

### New technologies in construction

With advancements in science and technology, the global construction industry is increasingly integrating innovative technologies, materials, processes, and methodologies to achieve comprehensive carbon reductions. In the context of construction-phase innovations, the International Organization for Standardization (ISO) mandates in *ISO 19,650: Building Information Modelling (BIM) Management* that BIM models must include carbon emission data fields, allowing for seamless integration with IoT devices. The American Society for Testing and Materials (ASTM), through *ASTM E1673: Standard Practice for Quality Control of Prefabricated Buildings*, requires that prefabricated component production adopt digital quality traceability systems, thereby enhancing precision and fostering intelligent carbon management in construction. Similarly, the European Norm (EN) mandates the digital recording of welding parameters in *EN 1090: Execution of Steel Structures and Aluminium Structures*. In Singapore, the Building and Construction Authority (BCA) incentivizes photovoltaic-integrated buildings with floor area ratio bonuses and requires rainwater systems to connect to smart water management platforms under its *Green Mark Scheme*, promoting clean energy and resource recycling technologies during construction. China’s *GB 50,300–2013: Unified Standard for Constructional Quality Acceptance of Building Engineering* also sets forth stringent requirements for green construction practices.

Through a systematic analysis of the emerging carbon-reduction technologies outlined in these standards, this study identifies 25 widely adopted and extensively implemented technologies for construction-phase carbon reduction (Table 1):

**Table 1:** Emerging technologies for construction-phase carbon reduction.

No	Emerging Technologies (Experimental group)	Traditional technologies (Control group)
1	Application technology of high-strength & high-performance concrete	Ordinary concrete (C30)
2	Application technology of high strength bar	Ordinary bar (HRB300)
3	Application technology of high strength steel	Ordinary steel (Q355)
4	High-strength bar straight thread connection technology	Electroslag pressure welding
5	Mechanical anchorage technology of bar	Flexural anchorage
6	Long auger hole pressure pile technology	Rotary drill hole mud wall protection drill pile technology
7	Combined aluminum alloy formwork technology	Wooden formwork
8	Combined ribbed plastic formwork technology	Wooden formwork
9	One time forming technology of concrete floor	Leveling layer construction in ground construction
10	Technology of no plastering on building walls	Common plaster in wall construction
11	Water collection & comprehensive utilization technology	Municipal water supplies
12	Construction waste reduction & resource utilization technology	Construction waste transportation and landfilling
13	Solar energy & Air energy utilization technology	Municipal electricity supplies
14	Application technology of steel bar welding mesh	Steel bar straightening, cutting, bending and welding on the construction site
15	Processing & distribution technology of molding steel bar products	Steel bar straightening, cutting, bending and welding on the construction site
16	Precast segmental box girder formwork technology	Field formwork technology
17	Prefabricated concrete shear wall structure technology	Cast-in-place concrete shear wall
18	Prefabricated concrete frame structure technology	Cast-in-place concrete frame structure
19	Concrete laminated floor technology	Cast-in-place floor technology
20	Precast concrete wall hanging panel technology	Cast-in-place concrete wall panels
21	Sandwich insulation wall panel technology	Large mold built-in insulation panel wall
22	Superimposed shear wall structure technology	Cast-in-place shear wall technology
23	Precast prestressed concrete member technology	Prestressed concrete component site construction technology
24	Factory production and processing technology of prefabricated components	Field prefabricated component construction technology
25	Steel structure residential application technology	Cast-in-place concrete structure residential application technology

It should be noted that, variations in building types (e.g., schools, residential buildings, gymnasiums), structural forms (e.g., masonry-concrete, reinforced concrete, steel structures), and project scales introduce substantial uncertainty in the selection and quantity of on-site construction techniques per square meter of GFA during the construction phase. To address this uncertainty, this study calculates carbon emission reductions based on the specific measurement units of each construction technique and incorporates the actual selection and consumption of these techniques within the project for computation.

However, the technologies listed in Table 1 cover diverse areas such as concrete, drilling, steel, formwork, water, electricity, and construction waste. Each material has distinct measurement units, making direct comparison of carbon reductions across technologies statistically insignificant. For instance, concrete is measured in 10 m^3^, steel in tons, formwork in 100 m^2^, water in m^3^, and electricity in kilowatt-hours (kW·h). To address this inconsistency in measurement units, a relative carbon reduction percentage is introduced to quantify the emission reduction benefits of each technology relative to its traditional counterpart.

### Methods

#### Carbon emission boundary determination

Establishing an appropriate boundary is crucial for accurate carbon emission analysis, as it defines emission responsibility^19^. This study focuses on on-site carbon emissions during the construction phase, corresponding to Module A5 (Construction-Installation Process) as defined in *ISO 21,930*. This module includes all carbon emissions directly generated from activities where materials and components are assembled into the final building structure through on-site processing, installation, and other construction operations. The key input elements are materials and energy, while the primary output is construction waste:

1. Material-related carbon emissions arise from the consumption of gases such as CO_2_ and C_2_H_2_ during on-site processing activities like electroslag pressure welding and CO_2_-shielded welding. This calculation accounts only for direct carbon emissions from material leakage or combustion at the construction site, distinct from the embodied carbon generated during the initial production phase.
2. Energy-related carbon emissions stem from the electricity, gasoline, and diesel consumed by construction machinery (e.g., elevators, cranes, excavators, water pumps) and temporary site lighting and office operations.
3. Emissions from construction waste result from transporting discarded materials, such as steel rebar, formwork, and muck, to centralized treatment facilities. This includes emissions from equipment used for both vertical (e.g., elevators) and horizontal transport (e.g., trucks).

Carbon emissions during the on-site construction phase are calculated as follows:

1 $$E = Em + Ee + Ew$$

where *E* is the total carbon emission of the construction technology, *E*_*m*_ is the carbon emission from material consumption, *E*_*e*_ is the carbon emission from equipment consumption, and *E*_*w*_ is the carbon emission from waste transportation.

#### Carbon emissions calculation

The formulas in this section are derived from the carbon emission calculation methodologies for Industrial Processes and Product Use (IPPU) in Volume 3 of the *2006 IPCC Guidelines for National Greenhouse Gas Inventories (2019 Refinement)*, as published by the Intergovernmental Panel on Climate Change (IPCC).

##### Carbon emission calculation from material consumption

Carbon emissions from material consumption in construction technology include those generated from water, electricity, CO₂ gas, acetylene gas, and other on-site materials. The formula is:

2 $$Em = \sum\limits_{i = 1}^{n} {mi*EFm,i}$$

In this equation, *n* represents the number of material types, *m*_*i*_ denotes the consumption of each material *i*, and *EF*_*m*_,_*i*_ is the carbon emission factor associated with material *i*. The types of materials consumed in the construction technology, along with their corresponding carbon emission factors, are provided in Online Appendix 1.

##### Carbon emission calculation from equipment use

Carbon emissions from equipment in construction technology primarily result from electricity or diesel consumption by various machines, such as concrete smoothers, welders, circular saws, cranes, and dump trucks. The formula is:

3 $$Ee = \sum\limits_{i = 1}^{n} {mi*Ci*EFe,i}$$

Here, *n* refers to the number of equipment types, *m*_*i*_ is the consumption values for each piece of equipment *i*, *C*_*i*_ is the energy consumption coefficient for equipment *i*, and *EF*_*e*_,_*i*_ is the carbon emission factor for the energy consumed by equipment *i*. Equipment types and their respective energy consumption levels are listed in Online Appendix 2, and the carbon emission factors for electricity and diesel are detailed in Online Appendix 1.

##### Carbon emission calculation from waste transportation

Waste transportation emissions are generated by moving waste materials—such as discarded formwork, channel steel, bolts, pipes, and support rods—from the construction site vertically to the ground and horizontally to recycling stations. The formula is:

4 $$Ew = \sum\limits_{i = 1}^{n} {mi*Vi*1.3*\left( {Hi*Ch*EFw,h + Si*Cs*EFw,s} \right)}$$

In this expression, *n* is the number of waste types, *m*_*i*_ represents the output of waste *i*, and *V*_*i*_ is the volume coefficient for waste *i*. The constant 1.3 accounts for the waste compression factor. *H*_*i*_ is the vertical transportation distance for waste *i*, *C*_*h*_ is the energy coefficient for the vertical transport equipment, and *EF*_*w,h*_ is the carbon emission factor for energy consumed during vertical transport. *S*_*i*_ is the horizontal transportation distance for waste *i*, *C*_*s*_ represents the energy coefficient for the horizontal transport equipment, and *EF*_*w,s*_ is the corresponding carbon emission factor. Online Appendix 3 details waste types and volume coefficients, while Online Appendices 1 and 2 list energy consumption and carbon emission factors.

## Results & discussion

### Analysis of carbon reduction pathways and classification

This study categorizes emerging construction-phase technologies into four groups based on their carbon reduction pathways:

1. High-strength and high-performance material technologies: These technologies reduce on-site material usage by enhancing material properties, thereby lowering construction-related carbon emissions.
2. Simplified construction process technologies: By integrating innovative materials and methods, these technologies streamline or eliminate traditional construction processes, reducing the need for equipment, minimizing material consumption, and curbing construction-related carbon emissions.
3. Renewable resources and waste reduction technologies: These technologies replace fossil fuels with renewable energy sources to lower grid electricity emissions, while waste recycling mitigates emissions associated with transportation and landfilling.
4. Prefabrication technologies: By transferring key processes (e.g., rebar tying, concrete pouring, component curing) to factory settings and shifting to on-site assembly, these technologies reduce energy-intensive operations and associated carbon emissions.

**Fig. 1:**
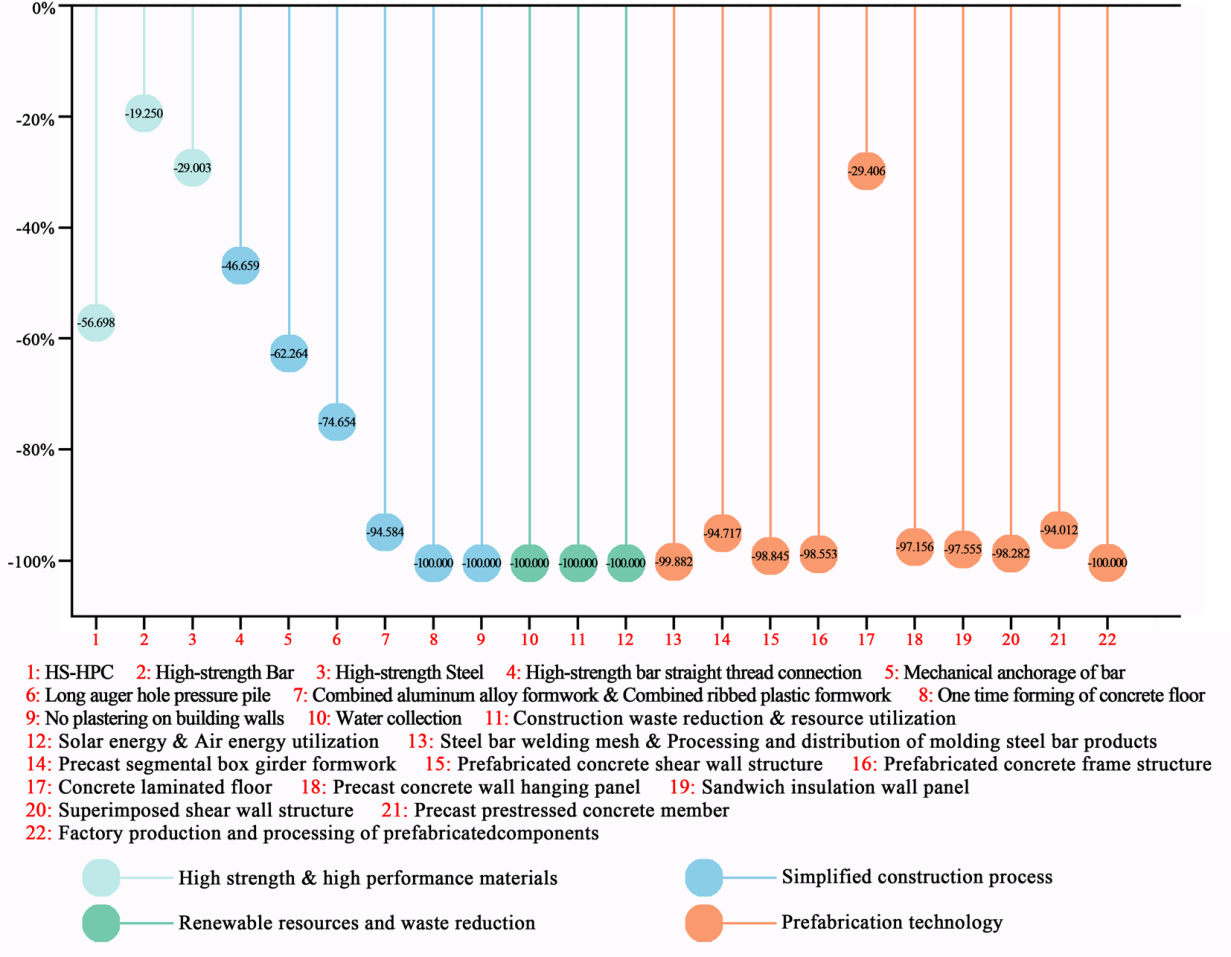
Carbon reduction percentage of emerging construction-phase technologies. (Drawn by Zhiping Liu, using Origin2024 and Adobe Photoshop 2023).

The findings, supported by calculated carbon reduction percentages (Fig. 1), reveal that renewable resources and waste reduction technologies offer the highest carbon reduction potential, achieving a 100% reduction in on-site emissions by utilizing clean energy sources such as solar energy and recycling zero-carbon materials like rainwater. Kang et al.^20^ also highlighted that advanced solar curtain wall systems can achieve significant carbon reductions. However, the implementation of these technologies requires additional devices for clean energy collection and waste regeneration, introducing supplementary embodied carbon emissions. Prefabrication technologies rank second, with concrete laminated floor technology showing a lower reduction rate, while other prefabrication technologies reduce emissions by over 90%. Bian et al.^21^ further confirmed the substantial carbon mitigation potential of prefabrication technologies throughout a building’s lifecycle. Simplified construction process technologies exhibit varying degrees of carbon reduction based on their specific impact on construction practices. Dou et al.^22^ also observed variations in the carbon reduction efficiency of different construction process simplification technologies in a decarbonization retrofit study of an office complex. Notably, high-strength bar straight-thread connection technology achieves the lowest carbon reduction at 47%, while one-time forming technology for concrete floors and no-plastering wall technology achieve 100% reductions at the construction site. Similar to renewable resources and waste reduction technologies, the 100% reduction in on-site emissions attributed to one-time forming technology for concrete floors and no-plastering wall technology is typically limited to the construction site. However, this achievement may inadvertently shift the carbon reduction burden upstream to companies involved in the prefabrication of floor slabs and wall panels, thereby increasing pressure on these enterprises to decarbonize. Despite this, the industrialized integrated prefabrication model still offers substantial carbon reduction advantages over traditional on-site construction methods. The factory environment enables highly efficient and precise manufacturing, minimizing material waste. Additionally, the controlled setting facilitates the implementation of stringent energy management practices and the adoption of more efficient technologies, resulting in lower carbon emissions during the production phase. The transfer of a significant portion of work from the unpredictable construction site to a controlled factory also leads to substantial reductions in construction duration, noise, dust, and on-site waste, further decreasing environmental impacts at the site. High-strength and high-performance material technologies exhibit the weakest carbon reduction effects, with the most effective technology reducing emissions by only 57%, while the others contribute reductions below 30%. These technologies do not directly reduce carbon-emitting activities or energy consumption during construction but minimize material usage on-site, resulting in indirect reductions in carbon emissions. Han et al.^23^ explored the carbon mitigation potential of high-performance concrete, demonstrating that enhanced concrete strength facilitates emission reduction by lowering material consumption.

The analysis and categorization of carbon reduction pathways for emerging construction-phase technologies not only support the achievement of short-term emission reduction goals but also foster synergistic carbon mitigation through the integration of multiple technologies. For instance, combining prefabrication technologies (which reduce on-site operations) with renewable energy utilization (which minimizes fossil fuel consumption) significantly enhances overall carbon reduction across the construction phase. Additionally, pathway analysis provides insights into the scenarios in which different technologies are most applicable and highlights cost-effectiveness variations, offering valuable guidance for technology selection.

### High-strength & high-performance material technologies

**Fig. 2:**
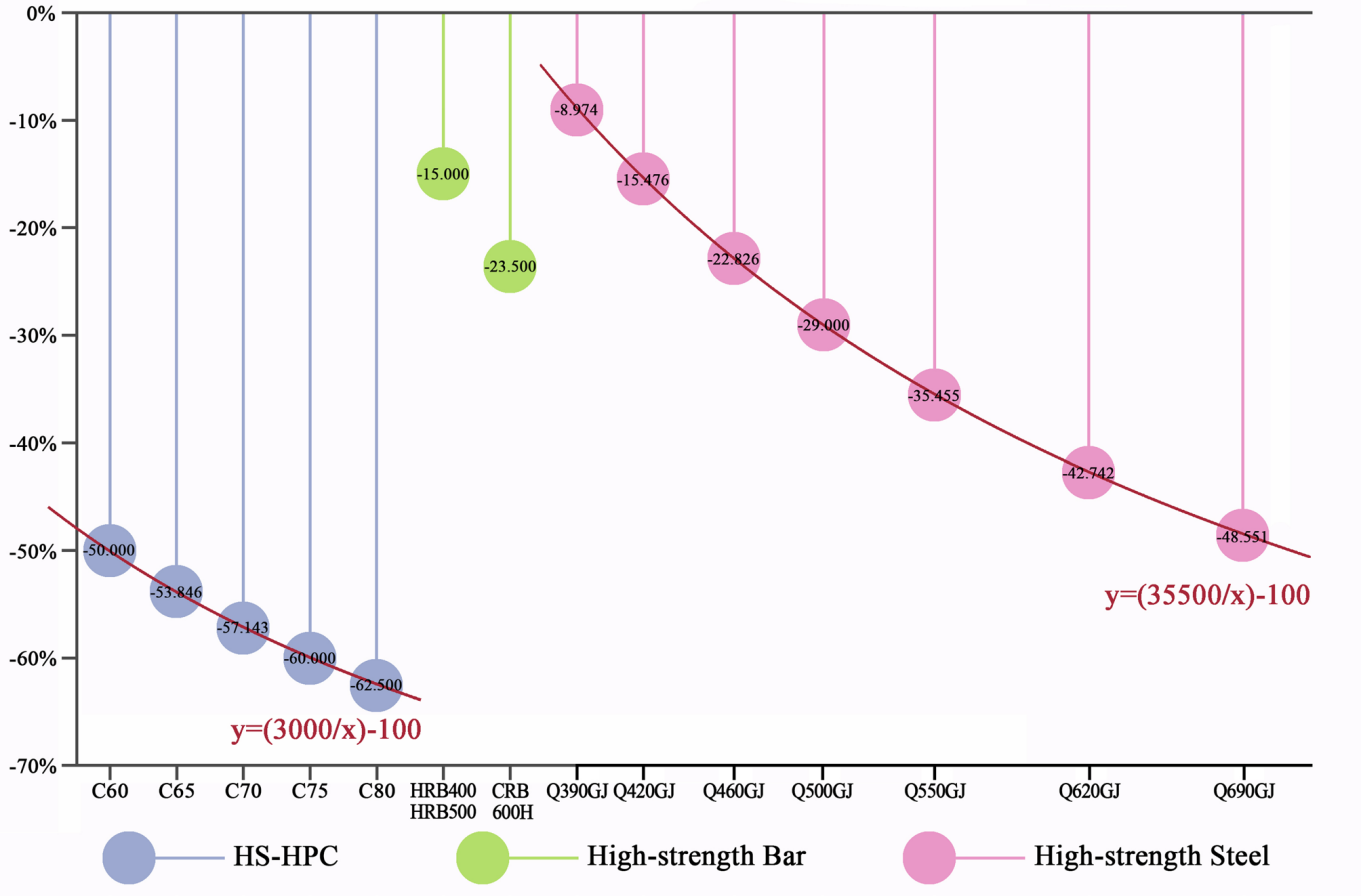
Carbon reduction percentage of high-strength & high-performance materials technologies. (Drawn by Zhiping Liu, using Origin2024 and Adobe Photoshop 2023).

In this category, the enhanced strength or performance of materials reduces the quantity needed for components with comparable performance, thereby decreasing the workload during the construction phase. As a result, carbon emissions from material and equipment consumption on-site are reduced (Fig. 2). Research by Kim et al.^24^ indicated that the use of high-strength steel can reduce steel consumption and lower CO_2_ emissions from both steel production and component manufacturing. Similarly, studies by Cho et al.^25^ highlighted the benefits of high-strength reinforcing bars in reducing CO_2_ emissions. While research by Thilakarathna et al.^26^ showed that high-strength concrete generates more carbon emissions during production than conventional concrete, its use significantly reduces overall concrete consumption. The resulting carbon reduction from the reduced material volume is more substantial, effectively lowering the overall carbon footprint. Detailed calculation results are provided in Online Appendix 5.

Overall, HS-HPC technology achieves the most significant carbon emission reduction, while high-strength bar application technology results in the smallest reduction. Our analysis further reveals that as material strength increases, the relative carbon emission reductions of these technologies decrease, although the rate of reduction gradually slows. In the case of HS-HPC and high-strength steel technologies, the relationship between material strength and carbon emission reduction follows an inverse proportional function.

High-strength and high-performance concrete (HS-HPC) technology demonstrates that, for the same component volume, beam and plate elements generate more carbon emissions than foundations, columns, and walls. This is due to concrete’s compressive strength, which contrasts with its tensile weakness. Beams and plates experience both compressive stress at the top and tensile stress at the bottom, whereas foundations, columns, and walls are subject only to compression. Consequently, beams and plates require more stringent maintenance during concrete curing, leading to higher water consumption and increased carbon emissions during construction. Similarly, elements such as staircases and after-pour belts, which have larger surface areas relative to their volume, also incur greater carbon emissions due to increased water usage on-site. Gao et al.^27^ also found that the carbon reduction efficiency of high-strength and high-performance concrete varies significantly depending on its application to different building components. (Table 2).

**Table 2:** Carbon reduction of HS-HPC technology in different building components.

		Unit: 10m^3^				
Project		HS-HPC technology				
		C60	C65	C70	C75	C80
Name		Carbon reduction (kgCO_2_)				
Foundation	Cushion layer	− 0.500	− 0.538	− 0.571	− 0.600	− 0.625
	Strip foundation	− 1.953	− 2.101	− 2.230	− 2.343	− 2.441
	Independent foundation	− 1.320	− 1.420	− 1.507	− 1.584	− 1.650
	Cup foundation	− 1.419	− 1.526	− 1.620	− 1.702	− 1.773
	Raft foundation	− 1.644	− 1.768	− 1.877	− 1.972	− 2.055
	Pile cap	− 1.871	− 2.013	− 2.136	− 2.245	− 2.338
	Equipment Foundation	− 1.417	− 1.524	− 1.618	− 1.700	− 1.771
Column	Rectangular column	− 0.410	− 0.441	− 0.468	− 0.491	− 0.512
	Structural column	− 1.809	− 1.946	− 2.065	− 2.170	− 2.261
	Special-shaped column	− 2.274	− 2.447	− 2.597	− 2.729	− 2.843
	Circular column	− 2.274	− 2.447	− 2.597	− 2.729	− 2.843
	Skew column	− 2.207	− 2.374	− 2.520	− 2.648	− 2.758
	Steel pipe column	− 2.286	− 2.460	− 2.611	− 2.743	− 2.858
	Steel reinforced concrete column	− 1.460	− 1.571	− 1.667	− 1.752	− 1.825
Beam	Foundation connecting beam	− 1.828	− 1.967	− 2.088	− 2.194	− 2.285
	Rectangular beam	− 2.844	− 3.060	− 3.247	− 3.412	− 3.554
	Special-shaped beam	− 2.874	− 3.092	− 3.282	− 3.449	− 3.593
	Cantilever beam	− 2.932	− 3.155	− 3.348	− 3.518	− 3.665
	Girth	− 3.199	− 3.442	− 3.653	− 3.838	− 3.998
	Lintel	− 3.363	− 3.618	− 3.840	− 4.035	− 4.203
	Arc & arched beam	− 4.232	− 4.553	− 4.832	− 5.078	− 5.289
	Skew beam	− 3.186	− 3.428	− 3.638	− 3.823	− 3.983
	Steel reinforced concrete beam	− 3.195	− 3.437	− 3.648	− 3.833	− 3.993
Wall	Straight wall	− 2.207	− 2.374	− 2.520	− 2.648	− 2.758
	Curved concrete wall	− 1.704	− 1.833	− 1.945	− 2.044	− 2.129
	Short-leg shear wall	− 1.743	− 1.875	− 1.990	− 2.091	− 2.178
	Retaining wall	− 1.642	− 1.767	− 1.875	− 1.970	− 2.052
	Elevator shaft wall straight wall	− 2.688	− 2.892	− 3.070	− 3.226	-3.360
	Climbing mold concrete wall	− 1.912	− 2.057	− 2.183	− 2.294	− 2.389
	Laminated slab concrete composite wall	− 2.383	− 2.564	− 2.721	− 2.860	− 2.979
	Large mold built − in insulation panel wall	− 1.751	− 1.884	− 1.999	− 2.101	− 2.188
Plate	Beamed plate	− 1.751	− 1.884	− 1.999	− 2.101	− 2.188
	Beamless plate	− 3.668	− 3.946	− 4.188	− 4.401	− 4.584
	Plain slab	− 3.858	− 4.151	− 4.406	− 4.630	− 4.823
	Arch plate	− 4.623	− 4.974	− 5.279	− 5.547	− 5.778
	Shell plate	− 3.593	− 3.866	− 4.103	− 4.312	− 4.491
	Composite hollow plate	− 6.247	− 6.721	− 7.134	− 7.496	− 7.808
	Inclined plate & slope roof plate	− 4.828	− 5.195	− 5.514	− 5.794	− 6.035
	Rail plate	− 7.241	− 7.791	− 8.269	− 8.689	− 9.051
	Bay window plate	− 1.191	− 1.281	− 1.360	− 1.429	− 1.488
	Hanging plate	− 4.744	− 5.105	− 5.418	− 5.693	− 5.930
	Pick eaves & gutter	− 5.334	− 5.739	− 6.091	− 6.401	− 6.668
	Awning plate	− 5.103	− 5.491	− 5.828	− 6.124	− 6.379
	Cantilever plate	− 5.359	− 5.766	− 6.119	− 6.430	− 6.698
	Balcony plate	− 5.853	− 6.297	− 6.684	− 7.023	− 7.316
	Patch seams between precast panels	− 6.352	− 6.835	− 7.254	− 7.622	-7.940
Stair (unit: 10m^2^)	Straight	− 4.315	− 4.642	− 4.927	− 5.177	− 5.393
	Arc	− 0.941	− 1.013	− 1.075	− 1.129	− 1.176
	Spiral	− 0.937	− 1.008	− 1.070	− 1.124	− 1.171
After-cast strip	Beam	− 0.893	− 0.961	− 1.020	− 1.072	− 1.116
	Plate	− 3.082	− 3.316	− 3.520	− 3.698	− 3.853
	Wall	− 4.171	− 4.487	− 4.763	− 5.005	− 5.213
	Raft foundation	− 2.572	− 2.767	− 2.937	− 3.086	− 3.215
Others	Water dispersion	− 2.406	− 2.589	− 2.748	− 2.887	− 3.008
	steps	− 1.938	− 2.085	− 2.213	− 2.326	− 2.423
	Stadium stand	− 0.245	− 0.263	− 0.279	− 0.293	− 0.306
	Trench	− 5.669	− 6.099	− 6.473	− 6.802	− 7.086
	Handrail & pressure top	− 3.344	− 3.598	− 3.819	− 4.013	− 4.180
	Small component	− 3.787	− 4.074	− 4.324	− 4.544	− 4.733

For high-strength bar application technologies, bars with diameters between 10 and 25 mm generate more carbon emissions than both thinner bars (less than 10 mm in diameter) and thicker bars (ranging from 25–40 mm). Thinner bars can be manually connected through binding, while thicker bars are mechanically connected using bolts or sleeves, avoiding the energy-intensive welding process, and thus reducing carbon emissions. Chang et al.^28^ also highlighted in their study on the embodied carbon emissions of steel structures that rebar with diameters of 10–25 mm incurs 20–30% higher per-unit carbon emissions when welded compared to those connected mechanically.

In high-strength steel application technologies, as the size of a single steel structure increases, so does the carbon emission intensity. Larger structures require more lifting equipment, which in turn raises the carbon emissions associated with equipment use. Additionally, factories and warehouses, typically featuring larger steel structures than high-rise buildings, exhibit higher carbon emission intensities. Gao et al.^27^ also demonstrated that larger structural components require more energy for lifting, resulting in increased emissions during construction.

### Simplified construction processes technologies

**Fig. 3:**
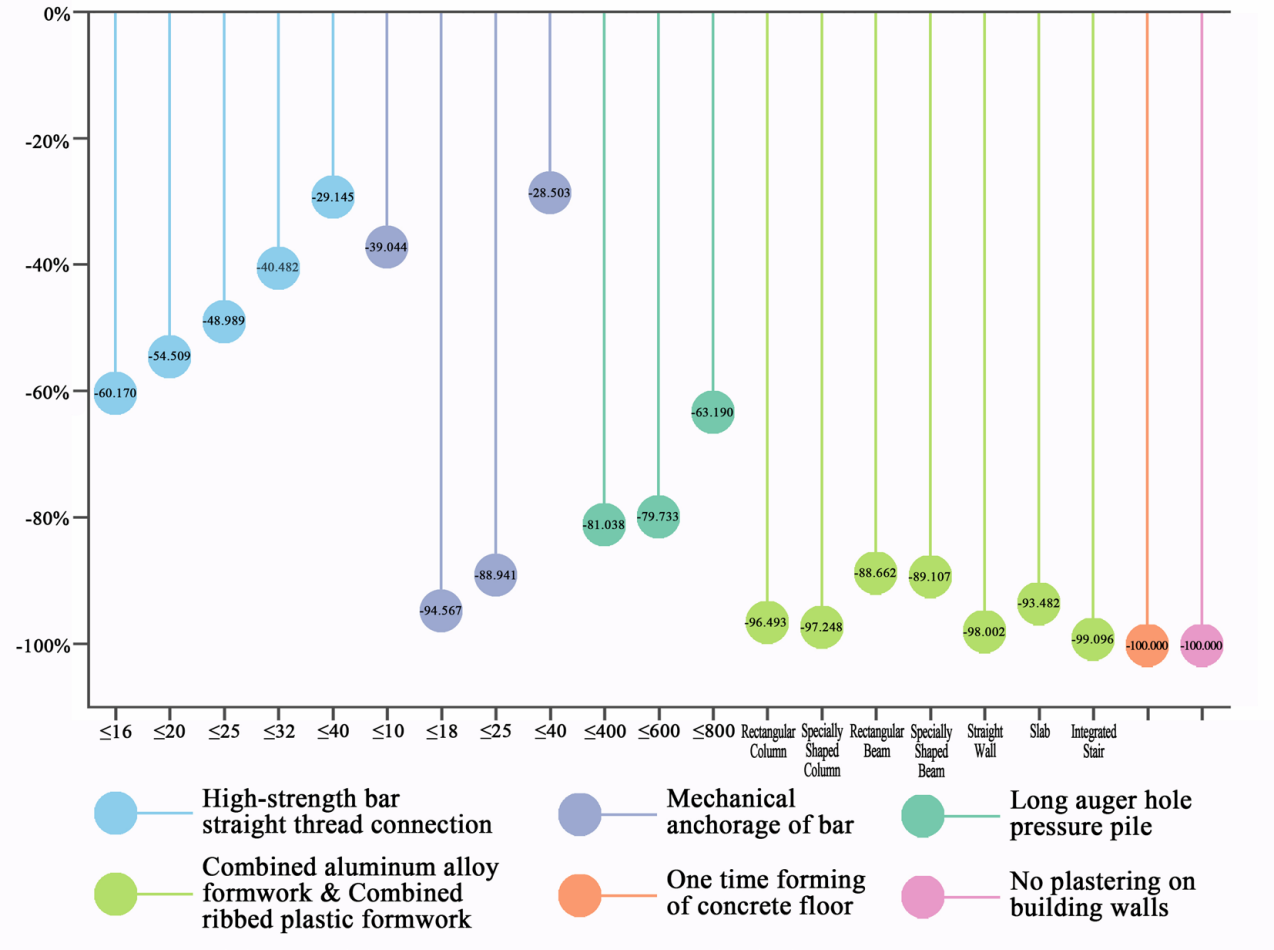
Carbon reduction percentage of simplified construction processes technologies. (Drawn by Zhiping Liu, using Origin2024 and Adobe Photoshop 2023).

This category of technologies reduces or eliminates several processes commonly involved in traditional construction, such as bar bending, welding, electrode drying, mud wall protection, formwork removal, and waste transfer, all of which significantly contribute to carbon reduction (Fig. 3). Shi et al.^8^ employed Building Information Modeling (BIM) technology to construct a building construction framework, also demonstrating that streamlining construction processes can reduce average energy consumption during the construction phase, thereby achieving carbon emission reduction. Detailed calculations are provided in Online Appendix 6.

Regarding relative carbon emission reductions, high-strength bar straight thread connection technology replaces the traditional welding process with a low-energy bolt sleeve wire machine, thereby reducing electricity consumption and eliminating the need for acetylene gas, which results in lower carbon emissions. It was also observed that, while traditional welding technology shows minimal variation in carbon emissions across different bar diameters, the high-strength bar straight thread connection technology leads to a rapid increase in emissions as bar diameters grow. Similarly, mechanical anchorage technology for bars eliminates the welding process, further reducing carbon emissions. In traditional anchoring methods, medium-thickness bars (10–25 mm in diameter) require welding, while thinner and thicker bars do not undergo this process, mirroring the pattern seen with high-strength bar applications discussed in Sect. "High-strength & high-performance material technologies". Consequently, mechanical anchorage technology has a more significant carbon reduction effect on medium-thickness bars. Widjaja et al.^29^ also demonstrated in their sustainability study of rebar mechanical connectors that replacing traditional welding with mechanical connectors eliminates welding gas emissions, thereby reducing the carbon footprint. Their research also highlighted varying carbon reduction rates depending on rebar diameters when using mechanical connectors.

In long auger hole pressure pile technology, the auger replaces the traditional rotary drill, which, while not offering energy savings, eliminates the need for mud wall protection, significantly reducing the demand for mud pumps and, consequently, lowering carbon emissions. However, for pile diameters exceeding 800 mm, mud pump collaboration remains necessary, limiting the carbon reduction potential of this technology for larger piles. A comparative case study by Sandanayake et al.^30^ also revealed that long auger hole pressure pile technology reduces carbon emission intensity per unit area in foundation construction.

Combined aluminum alloy and ribbed plastic formwork technologies replace traditional composite formwork with reusable aluminum or plastic forms, thereby reducing waste generation and, in turn, carbon emissions from waste transportation. However, their carbon reduction effectiveness is less pronounced for beams and plates, which require additional support rods during construction, thus increasing waste production. Zhang et al.^31^, in their low-carbon design research on concrete beams, also emphasized that the use of recyclable formwork systems further reduces carbon emissions.

One-time forming technology for concrete floors eliminates the floor leveling process, while no-plastering wall technology removes the need for wall plastering, preventing the carbon emissions associated with these activities. Thus, these technologies achieve a 100% reduction in carbon emissions at the construction site compared to traditional floor and wall construction methods, as these processes are entirely eliminated during construction. However, this may inadvertently transfer the carbon reduction responsibility upstream to prefabrication enterprises, intensifying their need to decarbonize.

In summary, technologies such as high-strength bar straight thread connection, mechanical anchorage for bars, and long auger hole pressure pile technology exhibit varying carbon reduction outcomes based on material diameters. Combined aluminum alloy and ribbed plastic formwork technologies demonstrate consistent carbon reduction across different construction components. One-time forming technology for concrete floors and no-plastering wall technology eliminate entire construction processes, resulting in a 100% reduction in carbon emissions from these tasks.

### Renewable resources & waste reduction technologies

**Fig. 4:**
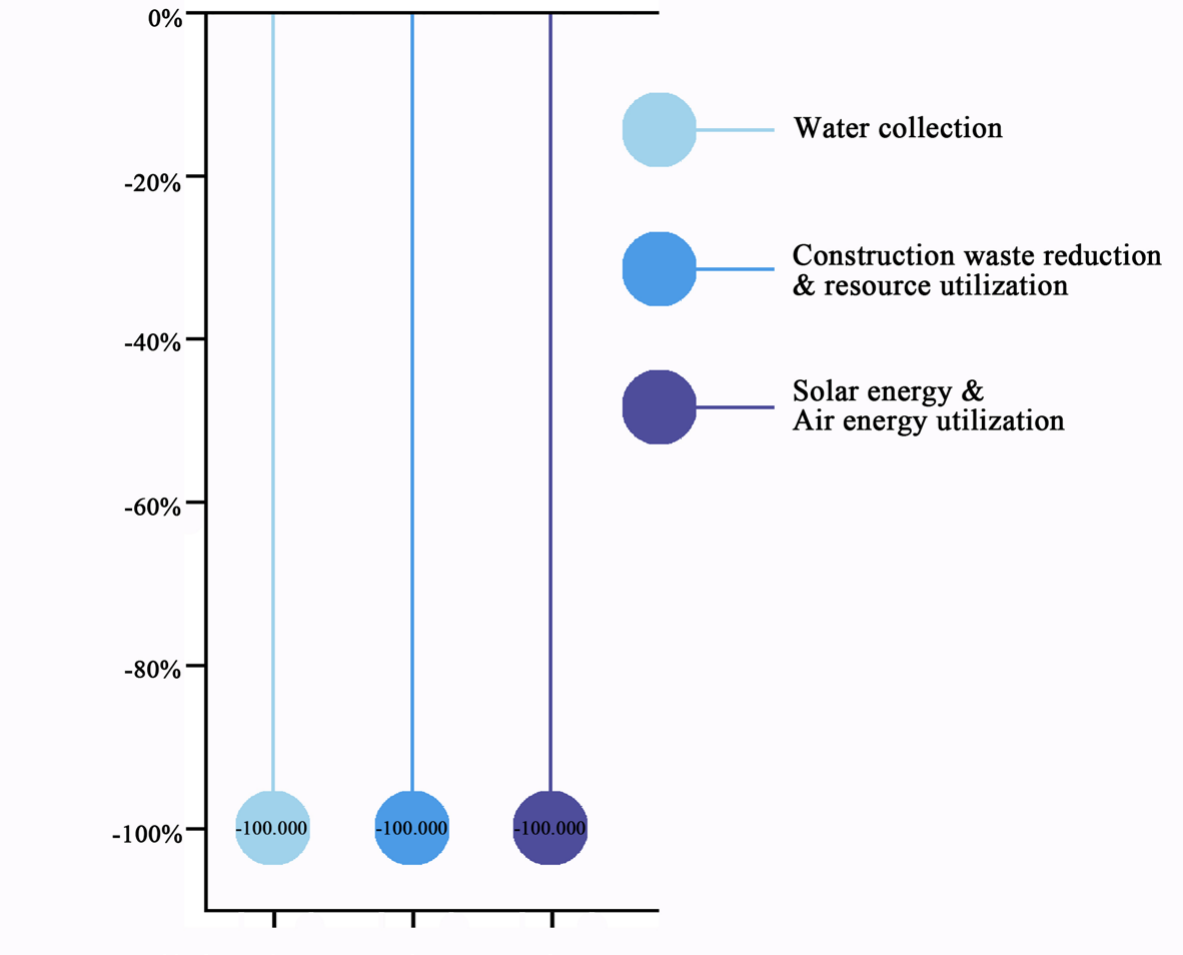
Carbon reduction percentage of renewable resources & waste reduction technologies. (Drawn by Zhiping Liu, using Origin2024 and Adobe Photoshop 2023).

The technologies in this category reduce carbon emissions associated with water supply consumption, waste transportation, and municipal power usage during the construction phase. Waste reduction eliminates the need for transportation, while the use of collected rainwater, solar energy, air energy, and other clean resources results in zero carbon emissions for all three technologies, yielding a relative carbon emission reduction of − 100% at the construction site. However, the implementation of these technologies requires additional infrastructure for clean energy collection and waste regeneration, introducing additional embodied carbon emissions. Similarly, research by Gu et al.^32^ highlighted that wastewater collection systems, while essential for urban life, significantly contribute to greenhouse gas emissions. Studies by Ma et al.^33^ emphasized that advanced energy technologies, such as renewable energy, integrated energy systems, and hybrid AC/DC power supply, can quickly, intuitively, and quantitatively achieve emission reductions. Furthermore, Wang et al.^34^ suggested that minimizing construction waste is critical for reaching carbon peak and carbon neutrality in the construction industry. Specific carbon reductions are calculated based on actual reductions in construction waste, rainwater recovery, and electricity consumption during the construction phase (Fig. 4). Detailed calculations are provided in Online Appendix 7.

### Prefabrication technologies

**Fig. 5:**
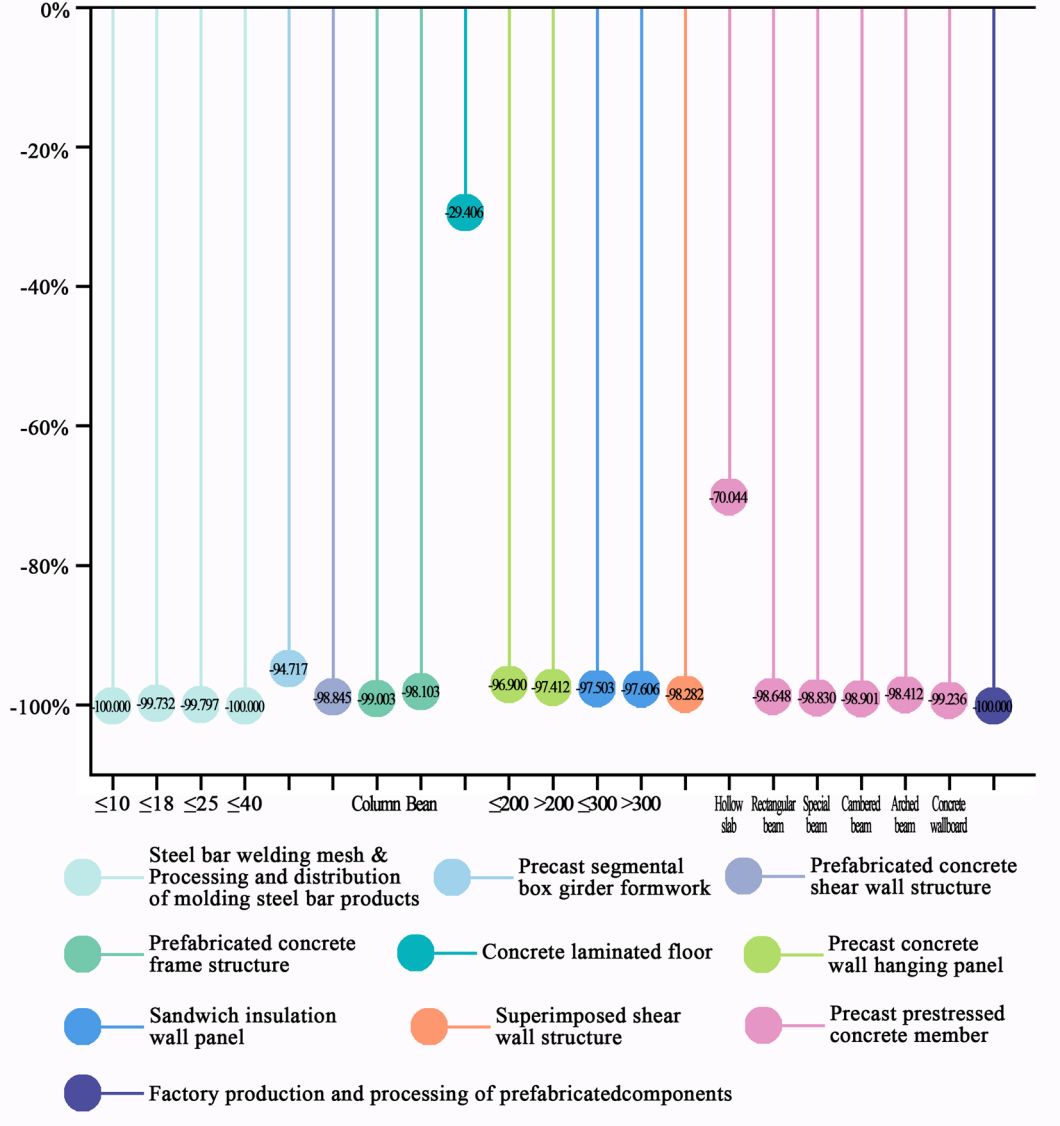
Carbon reduction percentage of prefabrication technologies. (Drawn by Zhiping Liu, using Origin2024 and Adobe Photoshop 2023).

These technologies directly install prefabricated materials on-site, replacing traditional processes such as support, binding, and pouring. This shift significantly reduces equipment consumption and waste emissions, resulting in carbon reductions during construction (Fig. 5). Teng et al.^35^ noted that employing prefabrication in the construction phase offers several benefits, including enhanced efficiency, reduced waste, and lower carbon emissions, though the extent of carbon reduction varies with different prefabrication techniques. Han et al.^36^ supported the use of prefabricated components as a viable pathway for reducing the construction industry’s carbon footprint, observing that carbon emissions during construction tend to decrease slightly as the prefabrication rate increases. Liu et al.^37^ proposed a two-stage greenhouse gas emission optimization method based on the EGVM model to improve emission management during the construction phase, enhancing decision-making for prefabricated and modular construction projects. Detailed calculations are available in Online Appendix 8.

Among these technologies, the factory production and processing of prefabricated components fully replace the on-site production process for traditional prefabricated components, with installation occurring directly at the construction site, leading to a relative carbon emission reduction of − 100%. Similar to one-time forming technology for concrete floors and no-plastering wall technology, this approach reduces carbon emissions at the construction site through advanced prefabrication in factories while simultaneously increasing pressure on upstream enterprises to reduce their own carbon emissions. Steel structure residential technology replaces traditional cast-in-place concrete structures with steel, simplifying the construction process. Steel structure construction primarily involves welding and lifting, which is less energy-intensive than traditional concrete construction that requires support, welding, pouring, curing, and mold removal. Additionally, on-site welding and lifting of steel structures have lower carbon emission coefficients, significantly reducing carbon emissions during the construction phase.

However, due to substantial differences in the quantity calculations between steel structures and cast-in-place concrete structures, and the considerable influence of the structural scheme, there is notable uncertainty in calculating the relative carbon emissions for steel structure residential application technology. This study calculates only the sub-components of steel structure residential and traditional cast-in-place concrete structure applications, meaning the total carbon emission reduction for steel structure technology must be analyzed on a project-specific basis.

Overall, the 12 technologies in this category demonstrate strong carbon reduction effects, most exceeding 95%. This is largely because the carbon emissions from these technologies stem primarily from the on-site installation of molding materials, greatly reducing emissions from raw material processing and waste transportation in traditional construction. Among these technologies, concrete laminated floor technology still requires bar welding, which consumes substantial electricity and generates carbon emissions, resulting in a relatively low carbon emission reduction percentage of only 29%. In precast prestressed concrete member technology, the carbon emission reduction for hollow slab construction is lower than for beam and wall slab construction, at just 70%. This is because hollow slab construction requires more welding and plate seam support, leading to higher electricity consumption and waste generation. Similarly, Mo et al.^38^ found in their carbon emission assessment of an office building that adopting prefabrication technology reduced construction-phase emissions by approximately 60% compared to traditional on-site methods.

### Emerging technology selection strategies

#### High-strength & high-performance material technologies

Designed for super high-rise buildings, long-span bridges, heavy-duty industrial plants, and public buildings with stringent seismic requirements. Examples include:

High-strength & high-performance concrete, which reduces column cross-sectional dimensions in core tubes and shear walls of super high-rises, increasing usable space while minimizing material usage and carbon emissions;

High-strength steel technology in long-span bridges, which reduces self-weight and enhances resistance to wind and seismic forces. Steel–concrete composite structures in cross-sea bridges further reduce material transport and construction energy demands;

High-strength bar technology in heavy-duty plants, which lowers reinforcement density and shortens installation time, reducing emissions during construction.

#### Simplified construction process technologies

Ideal for standardized residential buildings, urban complexes, and prefabricated construction projects. Examples include:

Combined aluminum alloy formwork technology, which eliminates plastering in standard residential floors, reducing template waste and labor;

High-strength bar straight thread connection technology, which replaces welding in high-rise office core tubes, reducing on-site energy consumption and exhaust emissions;

No-plastering wall technology, which eliminates wet processes, minimizing waste in commercial complexes;

Long auger hole pressure pile technology, which enhances efficiency in soft soil foundations, making it ideal for urban renewal projects with space limitations.

#### Renewable resources & waste reduction technologies

Critical for green-certified buildings, eco-parks, and water-sensitive regions. Examples include:

Building-integrated photovoltaic (BIPV) systems, which generate clean energy for airports and exhibition centers, reducing reliance on grid power;

Rainwater harvesting systems, which alleviate municipal water demand in schools and hospitals;

Construction waste recycling, which reduces transport distances and landfill pollution in urban renewal projects, supporting circular economy principles.

#### Prefabrication technologies

Optimal for modular housing, standardized factories, and rapid emergency facilities. Examples include:

Precast concrete components, which reduce energy consumption for on-site casting and formwork in batch housing projects;

Steel modular systems, which combine factory welding with on-site assembly, shortening construction timelines and reducing emissions;

Integrated prefabricated facades for skyscrapers, which minimize high-altitude risks and rework.

#### Comprehensive selection strategies

Aligning building functions, environmental constraints, and carbon reduction goals to maximize synergistic benefits. Examples include:

Urban Core Projects: Prioritize prefabrication and renewable technologies to reduce pollution and traffic disruption;

Ecologically Sensitive Zones: Focus on simplified processes and resource recycling to protect the environment;

Industrial/Infrastructure Projects: Combine high-strength materials and prefabrication to enhance durability and accelerate construction.

### Case verification

#### Project situation

**Fig. 6:**
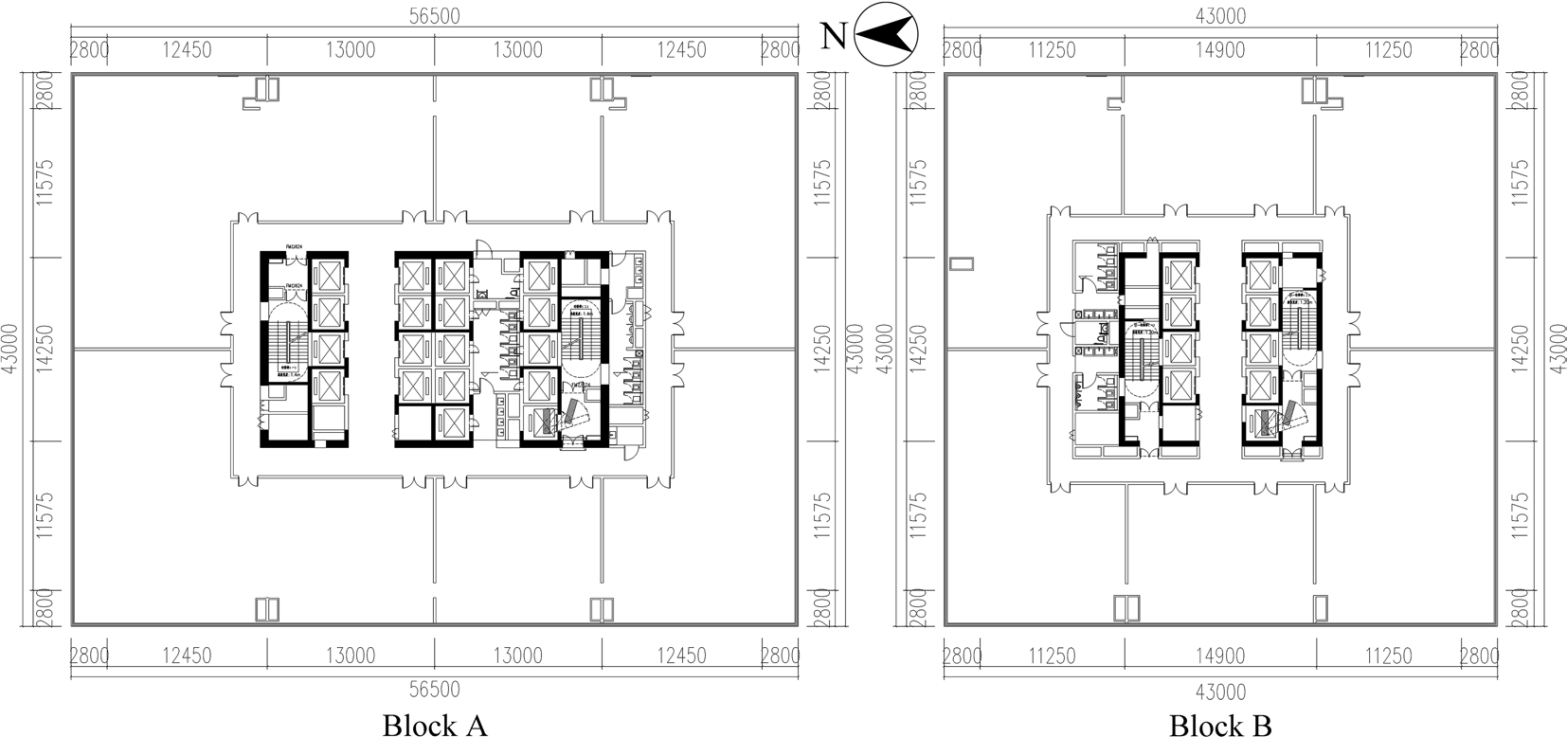
Schematic Architectural Plan of Xinyang CAZ Double Innovation Industrial Park. (Drawn by Zhiping Liu, using AutoCAD 2024 and Adobe Photoshop 2023).

The Xinyang CAZ Double Innovation Industrial Park is located in Yangshan District, Xinyang, China, on the west side of Xinyang High-Speed Railway East Station, with convenient transportation conditions. The project covers an area of about 39,700 m^2^, with a total construction area of approximately 217,500 m^2^, and the construction period spans about 550 days (1.5 years). The project consists of Block A, Block B, an underground garage, and the S1# building. Block A has an above-ground construction area of 92,422 m^2^, 37 floors, and a building height of 180 m. Block B has an above-ground construction area of 51,110 m^2^, 27 floors, and a building height of 135 m. The underground garage has a construction area of 64,000 m^2^ over two underground floors (Fig. 6).

Eight of the 25 carbon-efficient technologies were applied in the project (Table 3).

**Table 3:** Application of emerging technologies in Xinyang CAZ Double Innovation Industrial Park Project.

No	Category	Item	Application	Reduction capacity	Reduction (kg CO_2_)	Project reduction capacity (kg CO_2_ / 100 m^2^)
1	High strength & High performance material technologies	HS-HPC technology	C60 HS-HPC is used in the project, applied in the core tube shear wall and the basement steel concrete frame column, with a total amount of about 3,270 m^3^	0.59 kg CO_2_ / m^3^	1929.3	0.89
2		Application technology of high strength bar	Common hot-rolled ribbed steel bars (HRB) with yield strength of HRB400E and HRB500E are used in the project, mainly in the foundation and main structure, totaling 12,120 t	6.71 kg CO_2_ / t	81,326.0	37.39
3	Simplified construction processes technologies	High-strength bar straight thread connection technology	High-strength bars in the project are connected by straight thread connection technology, used in bars with diameters > 20 mm in the foundation and main structure, with 50,899 joints	0.32 kg CO_2_ / piece	16,287.7	7.49
4		Mechanical anchorage technology of bar	Mechanical anchoring technology of steel reinforcement is used, applying to the joint of steel-reinforced concrete columns and beams, with 552 anchoring plates used	2.19 kg CO_2_ / piece	1208.9	0.56
5		Combined aluminum alloy formwork technology	Aluminum alloy formwork is mainly used in the construction of the reinforced concrete core tube, with an application area of about 2,800 m^2^	0.33 kg CO_2_ / m^2^	914.8	0.42
6		Technology of no plastering on building walls	No plastering technology is applied on building walls, mainly in corridors and office partitions, reducing the plastering area by about 132,700 m^2^	0.06 kg CO _2_/ m^2^	7431.2	3.42
7	Renewable resources & waste reduction technologies	Construction waste reduction & resource utilization technology	New green construction technology, fine construction, and standardized construction measures are adopted to improve the reuse rate of wood formwork, reducing waste by1,954 m^3^	20.38 kg CO_2_ / m^3^	39,831.7	18.31
8		Solar energy & Air energy utilization Technology	Solar streetlights are used to illuminate the construction site, with 13 units installed, saving 2.08 kW·h/day of electricity	0.785 kg CO_2_ / kW·h	898.0	0.41
Total					149,827.6	68.89

#### Carbon emissions during the construction phase

**Fig. 7:**
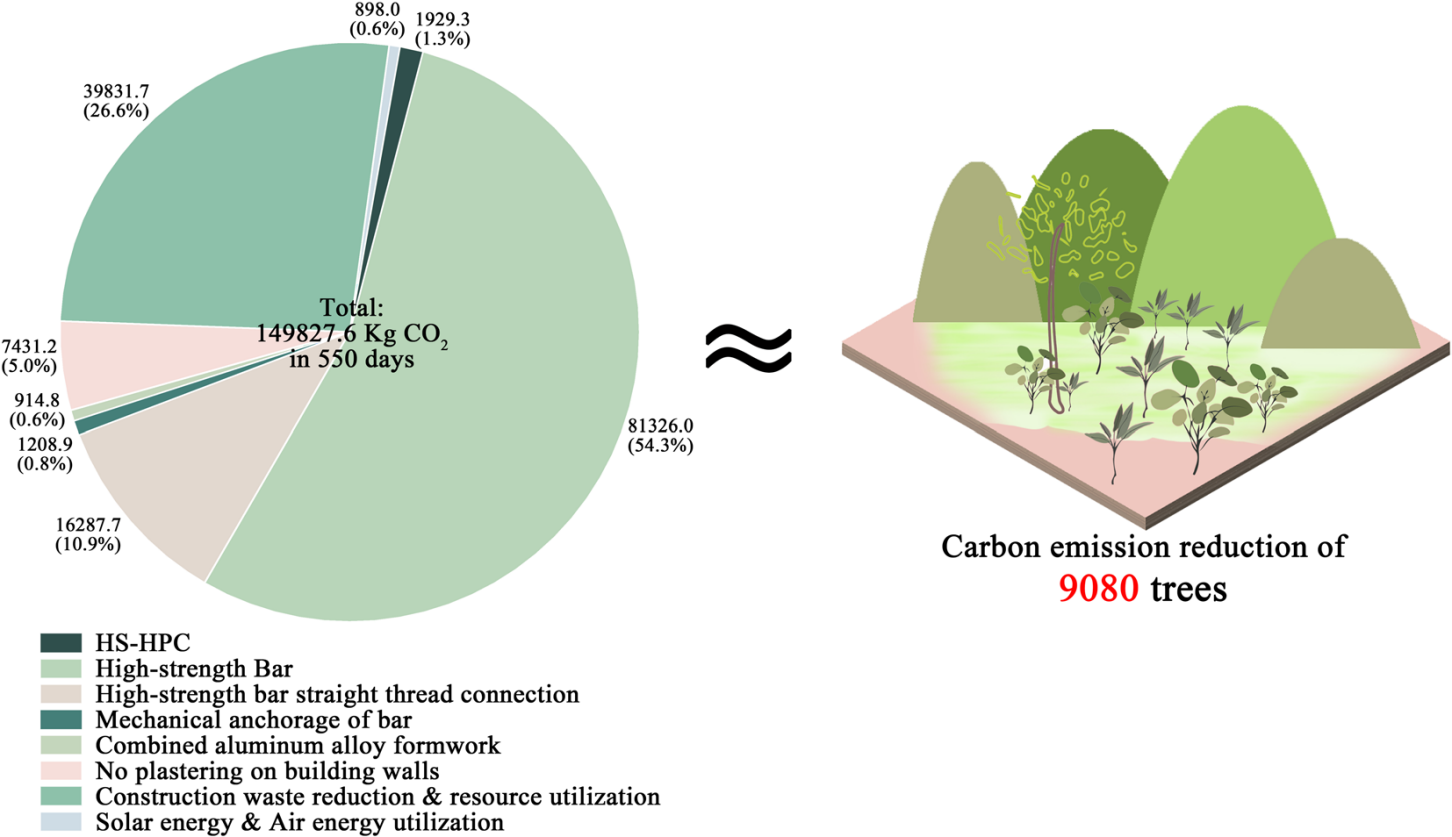
Carbon reduction benefits of emerging technologies in Xinyang CAZ Double Innovation Industrial Park Project. (Drawn by Zhiping Liu, using Origin2024 and Adobe Photoshop 2023).

Based on carbon reduction calculations for individual projects and the practical implementation of technologies, this study demonstrates that these innovations can achieve a 28.49% reduction in construction-phase carbon emissions (149,827.6 kg), equivalent to the carbon sequestration of 9,080 trees over the same period (Fig. 7). Among these technologies, high-strength bar technology resulted in the largest reduction, accounting for 54.3%, due to its application across all bars in the project, unlike other technologies, which were selectively applied. The second-largest reduction, 26.6%, was attributed to construction waste reduction and resource utilization technologies, owing to the high carbon reduction coefficient of waste transportation. High-strength bar straight thread connection technology and no-plastering wall technology also made significant contributions, representing 10.9% and 5.0% of the reduction, respectively, due to their extensive application. HS-HPC technology, mechanical anchorage technology, aluminum alloy formwork technology, and solar and air energy utilization technologies produced smaller reductions, collectively contributing only 3.3% due to less frequent use and lower carbon reduction coefficients.

## Conclusion

As a major contributor to global carbon emissions, the construction industry faces urgent decarbonization challenges throughout the building lifecycle. This study systematically assesses the carbon reduction potential of 25 emerging construction-phase technologies, highlighting significant variations in their emission reduction capabilities and pathways, thereby providing critical insights for technology selection:

1. Renewable Resources & Waste Reduction Technologies: These technologies achieve remarkable reduction in on-site carbon emissions by replacing fossil fuels with clean energy and eliminating off-site waste transportation, demonstrating the feasibility of full decarbonization. They are particularly well-suited for green-certified buildings, eco-parks, and water-sensitive projects.
2. Prefabrication Technologies: Emissions are reduced by over 90% through the transfer of on-site activities to controlled factory environments, showcasing their transformative potential in reshaping construction workflows. Ideal applications include modular housing, standardized factories, and rapid-deployment emergency facilities.
3. Simplified Construction Process Technologies: These technologies exhibit variable carbon reductions depending on the extent to which they eliminate energy-intensive processes such as formwork installation. They are best applied in standardized residential complexes, urban mixed-use developments, and prefabricated building auxiliary works.
4. High-strength & High-performance Material Technologies: These technologies offer limited carbon reductions (< 30%) by optimizing material quantities without addressing systemic energy or process inefficiencies. Their most effective applications are in supertall buildings, long-span bridges, heavy industrial plants, and seismically demanding public structures.
5. A real-world engineering case demonstrates that the integrated application of multiple technologies results in a 28.49% reduction in construction on-site carbon emissions, emphasizing the impact of strategic combinations of technologies.

The theoretical contribution of this study lies in the development of a “carbon reduction pathway—technology typology—reduction pathway” framework, which clarifies the prioritization of decarbonization technologies. In practical terms, it provides stakeholders with data-driven tools for decision-making, moving beyond vague sustainability goals. Policymakers can incentivize high-impact technologies through subsidies for emerging solutions and penalties for fossil fuel reliance, while enterprises must balance technology costs against emission reduction benefits, considering project-specific factors. However, several limitations of this study should be acknowledged. The analysis are orders of magnitude, which may vary from one project to another, and does not address critical challenges such as the scalability of emerging technologies, the logistical and carbon footprint implications of transporting prefabricated components across regions, or the varying effectiveness of policy instruments across different local governance contexts. Future research will focus on addressing these gaps, including full lifecycle cost assessments to evaluate scalability, the use of digital tools (e.g., BIM, AI) to optimize technology synergies and logistics, expanded dataset with greater variety to identify generalized principles governing technological interactions across diverse building typologies, and the evaluation of these technologies’ applicability and performance within diverse local policy frameworks.

## Supplementary Information

Below is the link to the electronic supplementary material.

Supplementary Material 1

## Data Availability

Data is provided within the manuscript or supplementary information files.

## References

[1] Lai, J. H. K., Yik, F. W. H. & Man, C. S. Carbon audit: A literature review and an empirical study on a hotel. *Facilities*10.1108/02632771211235233 (2012).

[2] UNEP. Sustainable building and climate change initiative. United Nations Environment Programme. http://www.unep.org/sbci/About SBCI/Background (accessed on 28 June 2024) (2014)

[3] Allouhi, A. et al. Energy consumption and efficiency in buildings: Current status and future trends. *J. Clean. Product.* **109**(DEC.16), 118–130. 10.1016/j.jclepro.2015.05.139 (2015).

[4] Akbarnezhad, A. & Xiao, J. Estimation and minimization of embodied carbon of buildings: A review. *Buildings* **7**(1), 5. 10.3390/buildings7010005 (2017).

[5] Kang, G., Kim, T., Kim, Y. W., Cho, H. & Kang, K. I. Statistical analysis of embodied carbon emission for building construction. *Energy Build.* **105**(OCT), 326–333. 10.1016/j.enbuild.2015.07.058 (2015).

[6] Pomponi, F. & Moncaster, A. Embodied carbon mitigation and reduction in the built environment - what does the evidence say?. *J. Environ. Manage.* **181**(Oct.1), 687–700. 10.1016/j.jenvman.2016.08.036 (2016). 27558830 10.1016/j.jenvman.2016.08.036

[7] Crawford, R. Life cycle assessment in the built environment. *Spon Press, London*10.4324/9780203868171 (2011).

[8] Shi, X. & Wang, L. Distribution Technique of green material list for high-rise building engineering in BIM technology. *Math. Problems Eng.* **2022**, 1–8. 10.1155/2022/6960596 (2022).

[9] Tae, K., Chang, C., Gil, K. & Hyoung, J. Analysis of CO_2_ emission characteristics of concrete used at construction sites. *Sustainability*10.3390/su8040348 (2016).

[10] Gu, L.G., Lu, S., Liu, C., Liu, J., & Ma, C. Development and application of super heavy gauge high-strength structural steel for high-rise buildings. IOP Publishing (Vol.242, pp.012062-). IOP Publishing. (2017)

[11] Dong, Y. H., Jaillon, L., Chu, P. & Poon, C. S. Comparing carbon emissions of precast and cast-in-situ construction methods: A case study of high-rise private building. *Constr. Build. Mater.* **99**, 39–53. 10.1016/j.conbuildmat.2015.08.145 (2015).

[12] Kaveh, A., Izadifard, R. A. & Mottaghi, L. Optimal design of planar RC frames considering CO_2_ emissions using ECOB, EVPS and PSO metaheuristic algorithms. *J. Build. Eng.* **28**, 101014. 10.1016/j.jobe.2019.101014 (2019).

[13] Salehian, S., Ismail, M. A. & Ariffin, A. R. M. Assessment on embodied energy of non-load bearing walls for office buildings. *Buildings*10.3390/buildings10040079 (2020).

[14] Jafarynasab, T., Monavari, S. M., Jozi, S. A. & Majedi, H. Assessment of carbon footprint in the construction phase of high-rise constructions in Tehran. *Int. J. Environ. Sci. Technol.* **17**(6), 3153–3164. 10.1007/s13762-019-02557-3 (2019).

[15] Tabrizikahou, A. & Nowotarski, P. Mitigating the energy consumption and the carbon emission in the building structures by optimization of the construction processes. *Energies* **14**(11), 3287. 10.3390/en14113287 (2021).

[16] Padilla-Rivera, A., Amor, B. & Blanchet, P. Evaluating the link between low carbon reductions strategies and its performance in the context of climate change: A carbon footprint of a wood-frame residential building in quebec, canada. *Mol. Diversity Preserv. Int.*10.3390/SU10082715 (2018).

[17] Monahan, J. & Powell, J. C. An embodied carbon and energy analysis of modern methods of construction in housing: A case study using a lifecycle assessment framework. *Energy Build.* **43**(1), 179–188. 10.1016/j.enbuild.2010.09.005 (2011).

[18] Ko, J. Carbon: Reducing the Footprint of the Construction Process. http://www.bis.gov.uk/policies/business-sectors/construction/sustainable-construction (accessed on 26 November 2021). (2010).

[19] Banerjee, S., Khan, M. A. & Husnain, M. I. U. Searching appropriate system boundary for accounting India’s emission inventory for the responsibility to reduce carbon emissions. *J. Environ. Manage.* **295**, 112907. 10.1016/j.jenvman.2021.112907 (2021). 34157542 10.1016/j.jenvman.2021.112907

[20] Kang, S. T., Park, J. H., Yuk, H., Yun, B. Y. & Kim, S. Advanced Trombe wall façade design for improving energy efficiency and greenhouse gas emissions in solar limited buildings. *Sol. Energy* **293**, 113492. 10.1016/j.solener.2025.113492 (2025).

[21] Bian, J. et al. Reducing carbon emissions from prefabricated decoration: A case study of residential buildings in China. *Buildings* **14**(2), 550. 10.3390/buildings14020550 (2024).

[22] Dou, Z., Jin, L., Chen, Y. & Huang, Z. Optimization of cost - carbon reduction: technology solution for existing office parks based on genetic algorithm. *Processes* **11**(8), 2452. 10.3390/pr11082452 (2023).

[23] Han, Y., Lin, R. & Wang, X. Carbon conversion technology for performance improvement and environmental benefits of ultra-high-performance concrete containing slag. *J. Mater. Res. Technol.* **21**, 2571–2583. 10.1016/j.jmrt.2022.10.075 (2022).

[24] Kim, D., Kim, J. & Chang, S. Material performance evaluation and super-tall building applicability of the 800 MPa high-strength steel plates for building structures. *Int. J. Steel Struct.* **14**(4), 889–900. 10.1007/s13296-014-1219-6 (2014).

[25] Cho, S. & Na, S. The reduction of CO_2_ emissions by application of high-strength reinforcing bars to three different structural systems in South Korea. *Sustainability* **9**(9), 1652. 10.3390/su9091652 (2017).

[26] Thilakarathna, P. S. M. et al. Embodied carbon analysis and benchmarking emissions of high and ultra-high strength concrete using machine learning algorithms. *J. Clean. Product.* **262**, 121281. 10.1016/j.jclepro.2020.121281 (2020).

[27] Gao, J. et al. Carbon emission analysis of RC core wall-steel frame structures. *Appl. Sci.* **14**(17), 7727. 10.3390/app14177727 (2024).

[28] Chang, H., Ma, S., Chiang, Y. & Lai, C. The effects of structural design alternatives on the embodied carbon emissions of steel buildings. *J. Build. Eng.* **99**, 111603. 10.1016/j.jobe.2024.111603 (2025).

[29] Widjaja, D. D., Khant, L. P., Kim, S. & Kim, K. Y. Optimization of rebar usage and sustainability based on special-length priority: A case study of mechanical couplers in diaphragm walls. *Sustainability* **16**(3), 1213. 10.3390/su16031213 (2024).

[30] Sandanayake, M., Zhang, G. & Setunge, S. Environmental emissions at foundation construction stage of buildings–Two case studies. *Build. Environ.* **95**, 189–198. 10.1016/j.buildenv.2015.09.002 (2016).

[31] Zhang, X. & Wang, F. Influence of parameter uncertainty on the low-carbon design optimization of reinforced concrete continuous beams. *Struct. Concrete: J. FIB*10.1002/suco.202100903 (2023).

[32] Gu, D. et al. Status of Research on greenhouse gas emissions from wastewater collection systems. *Water* **15**(14), 2512. 10.3390/w15142512 (2023).

[33] Ma, Y., Liu, W., Zhang, Z., Song, T. & Lv, Z. A practical method of carbon emission reduction ratio evaluation for expressway service area considering future infrastructures. *Energy Rep.* **9**, 1327–1337. 10.1016/j.egyr.2023.05.059 (2023).

[34] Wang, Z., Zhou, Y., Wang, T. & Zhao, N. Efficiency of construction waste and carbon reduction in the construction industry: based on improved three stage SBM-DEA model in China. *Eng. Construct. Arch. Manage.*10.1108/ECAM-10-2023-1088 (2024).

[35] Teng, Y., Li, K., Pan, W. & Ng, T. Reducing building life cycle carbon emissions through prefabrication: Evidence from and gaps in empirical studies. *Build. Environ.* **132**, 125–136. 10.1016/j.buildenv.2018.01.026 (2018).

[36] Han, Q., Chang, J., Liu, G. & Zhang, H. The carbon emission assessment of a building with different prefabrication rates in the construction stage. *Int. J. Environ. Res. Public Health* **19**(4), 2366. 10.3390/ijerph19042366 (2022). 35206554 10.3390/ijerph19042366 PMC8872307

[37] Liu, G., Huang, R., Li, K., Shrestha, A. & Fu, X. greenhouse gas emissions management in prefabrication and modular construction based on earned value management. *J. Construct. Eng. Manage.*10.1061/(ASCE)CO.1943-7862.0002268 (2022).

[38] Mo, Z. et al. An empirical study of carbon emission calculation in the production and construction phase of a prefabricated office building from Zhejiang China. *Buildings* **13**(1), 53. 10.3390/buildings13010053 (2022).

