# Supplementary material for: Carbon reduction potential and selection strategies of emerging construction-phase technologies
Source: Sci Rep. 2026 Feb 9;16:7863. doi: 10.1038/s41598-026-39122-1 (PMC12953918; doi:10.1038/s41598-026-39122-1)
Supplement: Supplementary file 1 — Supplementary Material 1 [file 41598_2026_39122_MOESM1_ESM.docx]

**Supplementary Materials**

**Appendix 1.** Carbon emission activity coefficient of material consumption

**Appendix 2.** Energy consumption per machine-team

**Appendix 3.** Volume coefficient of construction waste

**Appendix 4.** Steel bar and formwork consumption in cast-in-place concrete component construction (unit: 10 m^3^)

**Appendix 5.** Carbon emissions of high-strength & high-performance material technologies

**Appendix 6.** Carbon emissions of simplified construction processes technologies

**Appendix 7.** Carbon emissions of renewable resources & waste reduction technologies

**Appendix 8.** Carbon emissions of prefabrication technologies

**Appendix 1. Carbon emission activity coefficient of material consumption**[^[[1]](#footnote-1)^]

1. Water, 0.91 kg CO_2_ / m^3^.
2. Electricity, 0.785 kg CO_2_ / kW·h.
3. Diesel oil, calorific value 42652 kJ/kg, unit calorific value carbon emission coefficient 72.59 t CO_2_ / TJ, after calculation, diesel oil carbon emission coefficient is 3.096 kg CO_2_ / kg.
4. CO_2_ gas, the molar volume of carbon dioxide gas under standard atmospheric pressure is 22.4 L / mol, and the molar mass of carbon dioxide gas is 44 g / mol. By calculation, carbon dioxide gas under standard atmospheric pressure is 1.946 kg / m^3^.
5. C_2_H_2_ gas, carbon emission 3.96 kg CO_2_ / m^3^.

**Appendix 2. Energy consumption per machine-team[^[[2]](#footnote-2)^]**

1. Concrete smoothing machine, electricity consumption 23.14 kW·h / team.
2. Dry mortar tank mixer, electricity consumption 28.51 kW·h / team.
3. Mortar mixer 200 L, electricity consumption 8.61 kW·h / team.
4. Steel bar straightener 40 mm, electricity consumption 11.90 kW ·h / team.
5. Steel bar cutting machine 40 mm, electricity consumption 32.10 kW ·h / team.
6. Steel bar bending machine 40 mm, electricity consumption 12.80 kW ·h / team.
7. Dc arc welding machine 32 kV·A, electricity consumption 93.60 kW·h / team.
8. Ac arc welding machine 32 kV·A, electricity consumption 96.53 kW ·h / team.
9. CO_2_ gas shielded welding machine 500 A, electricity consumption 54.26 kW·h / team.
10. Butt welding machine 75 kV·A, electricity consumption 122.00 kW ·h / team.
11. Electroslag welding machine 1000 A, electricity consumption 147.00 kW ·h / team.
12. Voltage welding machine, electricity consumption 43.10 kW·h / team.
13. Welding rod drying box 45×35×45 (cm^3^), electricity consumption 6.70 kW·h / team.
14. Electric single-barrel slow winch 50 kN, electricity consumption 33.60 kW·h / team.
15. Prestressed steel tensile machine 650 kN, electricity consumption 17.25 kW·h / team.
16. Woodworking circular saw, electricity consumption 24.00 kW ·h / team.
17. Woodworking single side press planer 600 mm, electricity consumption 28.60 kW·h / team.
18. Bolt sleeve wire machine, electricity consumption 25.00 kW ·h / team.
19. Single cage construction elevator 1 t 75 m, electricity consumption 42.32 kW ·h / team.
20. Rotary drill 800 mm, electricity consumption 142.25 kW ·h / shift.
21. Rotary drill 1000 mm, electricity consumption 163.72 kW ·h / team.
22. Rotary drill 1500 mm, electricity consumption 190.72 kW·h / team.
23. Auger 400 mm, electricity consumption 123.48 kW ·h / team.
24. Auger 600 mm, electricity consumption 181.27 kW ·h / team.
25. Auger 800 mm, electricity consumption 203.65 kW ·h / team.
26. Mud pump 100 mm, electricity consumption 234.60 kW·h / team.
27. Truck crane 20 t, diesel oil consumption 38.41 kg / team.
28. Truck crane 40 t, diesel oil consumption 48.52 kg / team.
29. Crawler crane 25t, diesel oil consumption 36.98 kg / team.
30. Crawler crane 50 t, diesel consumption 44.03 kg / team.
31. Wheeled loader 0.5 m^3^, diesel oil consumption 46.71 kg / team.
32. Dump truck 8 t, diesel oil consumption 40.93 kg / team.

**Appendix 3. Volume coefficient of construction waste**

1. Thickness of composite formwork is 0.018 m and 0.021 m, which are common.
2. Steel support and accessories, galvanized channel steel, tension bolt, steel parts (comprehensive), steel back, metal structure iron parts and other steel materials, the density is 7850 kg / m^3^.
3. Plastic hard pipe *φ*20 cross-sectional area is 0.0003 m^2^.
4. Semi-hard plastic pipe *φ*32 cross-sectional area is 0.0008 m^2^.
5. Supporting rod *φ*48×3.5 length is 3 m, and the volume is 0.054 m^3^ / set.
6. Round nails, pin pieces, sporadic fixtures, pull pieces, hexagonal bolts with nuts (comprehensive), galvanized iron wire *φ*0.7, steel wire rope, pull hoop connectors, etc., because in the construction process has been embedded in the template or plate square materials, so the volume is not accounted for separately.
7. Pad iron, PE rod, embedded iron parts and positioning steel plate are all left inside the wall after installation, so they are not included in the external volume of construction waste.
8. Aluminum formwork, shaped steel formwork, etc. are not included in the external volume of construction waste because they can be recycled.
9. The crushing rate of the volume of construction waste is 1.3, according to the provisions of the Consumption of housing construction and decoration engineering (TY01-31-2021) that “construction waste is transported according to the virtual square volume”.
10. The carbon emission of construction waste external transportation is calculated according to the vertical transportation distance within 18 m and the horizontal transportation distance within 5 km, and taken as 203.847 kg CO_2_ / 10m^3^.

**Appendix 4. Steel bar[^[[3]](#footnote-3)^] and formwork[^[[4]](#footnote-4)^] consumption in cast-in-place concrete component construction (unit: 10 m^3^)**

1. Column: steel bar 1.731 t, formwork 96.618 m^2^.
2. Rectangular column: steel bar 1.731 t, formwork 96.618 m^2^.
3. Structural column: steel bar 1.731 t, formwork 99.900 m^2^。.
4. Special-shaped column: steel bar 0.564 t, formwork 100.200m^2^.
5. Circular column: steel bar 1.805 t, formwork 75.019 m^2^.
6. Beam: steel bar 1.858 t, formwork 91.241 m^2^.
7. Rectangular beam: steel bar 1.858 t, formwork 91.241 m^2^.
8. Special-shaped beam: steel bar 1.998 t, formwork 89.526 m^2^.
9. Girth: steel bar 0.949 t, formwork 82.034 m^2^.
10. Lintel: steel bar 1.172 t, formwork 135.501 m^2^.
11. Curved beam: steel bar 1.468 t, formwork 77.700 m^2^.
12. Arched beam: steel bar 2.081 t, formwork 89.127 m^2^.
13. Skew beam: steel bar 1.314 t, formwork 91.241 m^2^.
14. Prestressed rectangular beam: steel bar 1.858 t, formwork 91.241 m^2^.
15. Prestressed special-shaped beam: steel bar 1.998 t, formwork 89.526 m^2^.
16. Prestressed curved beam: steel bar 2.081 t, formwork 89.127 m^2^.
17. Prestressed Aarched beam: steel bar 1.468 t, formwork 77.700 m^2^.
18. Shear wall: steel bar 1.827 t, formwork 94.967 m^2^.
19. Large mold built-in insulation panel wall: steel bar 1.026 t, formwork 94.967 m^2^.
20. Prestressed wall: steel bar 1.300 t, formwork 94.967 m^2^.
21. Floor plate: steel bar 1.258 t, formwork 78.989 m^2^.
22. Hanging plate: steel bar 0.819 t, formwork 176.678 m^2^.
23. Prestressed hollow plate: steel bar 1.276 t, formwork 78.989 m^2^.
24. Field prefabricated lintel: steel bar 0.569 t, formwork 124.533 m^2^.
25. Field prefabricated trench cover: steel bar 0.569 t, formwork 124.533 m^2^.
26. Field prefabricated plate: steel bar 0.569 t, formwork 70.771 m^2^.
27. Field prefabricated overhead heat shield: steel bar 0.569 t, formwork 80 m^2^.
28. Field prefabricated hollow lattice: steel bar 0.569 t, formwork 1052.632 m^2^.
29. Field prefabricated small component: steel bar 0.569 t, formwork 210.526 m^2^.

**Appendix 5. Carbon emissions of high-strength & high-performance material technologies**

1. **Application technology of high-strength & high-performance concrete**

High-strength and high-performance concrete (HS-HPC) is a concrete with high strength (generally no less than C60) and has high workability, volume stability and durability. It is mostly used for the bottom columns, walls and large-span beams of high-rise buildings, which can reduce the section size of the components, thus reducing the amount of concrete used in the construction process. Affected by the reduction in the amount of concrete, the consumption of materials and machinery required in the construction process is also reduced, leading to a carbon reduction. When the pressure value of the member is unchanged, the volume of the compression member decreases with the increase of the compressive strength of the concrete, that is, the amount of concrete decreases.

The formula for calculating the pressure value of the concrete member is as follows (**Formula 1**):

$F=p\times S$ (**Formula 1**)

Where: *F* is the pressure on the component; *p* is the compressive strength of concrete; *S* is the cross-sectional area of the component.

In a certain project, the pressure *F* received by the compression member is certain, so the following mathematical relationship exists when using concrete of different strengths (**Formula 2**):

 (**Formula 2**)

At the same time, the height of the member *h* is also certain, so we can get the following mathematical relationship between the amount of concrete *V* and its compressive strength *p* (**Formula 3**):

 (**Formula 3**)

So far, we get the relationship between the amount of concrete and its strength grade (**Table 5.1.1**):

**Table 5.1.1.** Relations between concrete consumption and their compressive strength grades

|  | Ordinary performance concrete | | | | | | | | | High performance concrete | | | | |
| --- | --- | --- | --- | --- | --- | --- | --- | --- | --- | --- | --- | --- | --- | --- |
| Compressive strength grades | C15 | C20 | C25 | C30 | C35 | C40 | C45 | C50 | C55 | C60 | C65 | C70 | C75 | C80 |
| Compressive strength | 15 | 20 | 25 | 30 | 35 | 40 | 45 | 50 | 55 | 60 | 65 | 70 | 75 | 80 |
| Relative consumption | 1.000 | 0.500 | 0.200 | 0.000 | -0.14 | -0.250 | -0.333 | -0.400 | -0.455 | -0.500 | -0.538 | -0.571 | -0.6 | -0.625 |

Affected by the reduction in the amount of concrete, the consumption of materials and machinery required in the construction process is also reduced, which leads to a carbon reduction. Refer to *Building construction and decoration engineering consumption* (TY 01-31-2021) (hereiafter referred to as *BC&DEC*), it is known that the engineering consumption and carbon emissions required for concrete construction at each part (**Table 5.1.2 - 5.1.9**) :

**Table 5.1.2.** Consumption and carbon emissions of cast-in-place concrete foundation works

Unit: 10m^3^

| Project | | | Cushion layer | Strip foundation | | Independent foundation | | Cup foundation | Raft foundation | | Pile cap | Equipment foundation (unit: m^3^) | |
| --- | --- | --- | --- | --- | --- | --- | --- | --- | --- | --- | --- | --- | --- |
|  |  |  |  | Rubble concrete | Concrete | Rubble concrete | Concrete |  | Beamed type | Beamless type |  | ≤5 | ≤20 |
| Name | | Unit | Consumption / emissions | | | | | | | | | | |
| Material | Water | m^3^ | 3.950 | 0.930 | 1.009 | 1.091 | 1.125 | 1.200 | 1.339 | 1.520 | 1.125 | 1.115 | 0.900 |
|  | Electricity | kW·h | 2.310 | 1.980 | 2.310 | 1.980 | 2.310 | 2.310 | 2.310 | 2.310 | 2.310 | 2.310 | — |
| Machine | Concrete smoothing machine | Team | — | — | — | — | — | — | 0.035 | 0.030 | — | — | — |
| Carbon emission | | Kg CO_2_ | 5.408 | 2.401 | 2.732 | 2.547 | 2.837 | 2.905 | 3.668 | 3.741 | 2.837 | 2.828 | 0.819 |

**Table 5.1.3.** Consumption and carbon emissions of cast-in-place concrete column works

Unit: 10m^3^

| Project | | | Rectangular column | Structural column | Special-shaped column | Circular column | Skew column | Steel pipe column | Steel reinforced concrete column |
| --- | --- | --- | --- | --- | --- | --- | --- | --- | --- |
| Name | | Unit | Consumption / emissions | | | | | | |
| Material | Water | m^3^ | 0.774 | 1.789 | 1.789 | 1.658 | 1.789 | — | 0.774 |
|  | Electricity | kW·h | 3.710 | 3.720 | 3.720 | 3.700 | 3.750 | 3.720 | 3.760 |
| Carbon emission | | kg CO_2_ | 3.617 | 4.548 | 4.548 | 4.413 | 4.572 | 2.920 | 3.656 |

**Table 5.1.4.** Consumption and carbon emissions of cast-in-place concrete beam works

Unit: 10m^3^

| Project | | | Foundation connecting beam | Rectangular beam | Special-shaped beam | Cantilever beam | Girth | Lintel | Curved & arched beam | Skew beam | Steel reinforced concrete beam |
| --- | --- | --- | --- | --- | --- | --- | --- | --- | --- | --- | --- |
| Name | | Unit | Consumption / emissions | | | | | | | | |
| Material | Water | m^3^ | 3.040 | 3.090 | 3.200 | 3.795 | 4.640 | 6.065 | 3.759 | 3.795 | 3.362 |
|  | Electricity | kW·h | 3.720 | 3.740 | 3.760 | 3.750 | 3.188 | 3.750 | 3.760 | 3.740 | 3.760 |
| Carbon emission | | kg CO_2_ | 5.687 | 5.748 | 5.864 | 6.397 | 6.725 | 8.463 | 6.372 | 6.389 | 6.011 |

**Table 5.1.5.** Consumption and carbon emissions of cast-in-place concrete wall works

Unit: 10m^3^

| Project | | | Straight wall | | Curved concrete wall | Short-leg shear wall | Retaining wall (gravity type) | | Retaining wall (thin-walled) | Elevator shaft straight wall | Climbing mold concrete wall | Laminated slab concrete composite wall | Large mold built-in insulation panel wall |
| --- | --- | --- | --- | --- | --- | --- | --- | --- | --- | --- | --- | --- | --- |
|  |  |  | Rubble concrete | Concrete |  |  | Rubble concrete | Concrete | Concrete |  |  |  |  |
| Name | | Unit | Consumption / emissions | | | | | | | | | | |
| Material | Water | m^3^ | 0.454 | 0.587 | 0.672 | 0.587 | 0.534 | 0.690 | 2.690 | 0.759 | 1.450 | 0.690 | 0.690 |
|  | Electricity | kW·h | 3.060 | 3.660 | 3.660 | 3.730 | 3.060 | 3.660 | 3.730 | 3.990 | 4.390 | 3.660 | 3.660 |
| Carbon emission | | kg CO_2_ | 2.815 | 3.407 | 3.485 | 3.462 | 2.888 | 3.501 | 5.376 | 3.823 | 4.766 | 3.501 | 3.501 |

**Table 5.1.6.** Consumption and carbon emissions of cast-in-place concrete plate works

Unit: 10m^3^

| Project | | | Beamed plate | Beamless plate | Plain slab | Arched plate | Shell plate | Composite hollow plate | Skew plate & slope roof plate | Rail plate | Bay window plate | Hanging plate | Pick eaves & gutter | Awning plate | Cantilever plate | Balcony plate | Patch seams between precast panels |
| --- | --- | --- | --- | --- | --- | --- | --- | --- | --- | --- | --- | --- | --- | --- | --- | --- | --- |
| Name | | Unit | Consumption / emissions | | | | | | | | | | | | | | |
| Material | Water | m^3^ | 2.595 | 3.023 | 4.104 | 1.625 | 6.675 | 3.948 | 8.860 | 2.617 | 5.250 | 3.010 | 6.040 | 7.300 | 7.687 | 9.380 | 7.878 |
|  | Electricity | kW·h | 3.790 | 3.780 | 3.780 | 3.800 | 3.780 | 3.790 | 3.780 | — | 6.000 | 10.100 | 6.000 | 5.190 | 6.000 | 5.310 | 1.860 |
| Machine | Concrete smoothing machine | Team | 0.110 | 0.110 | 0.140 | 0.150 | 0.190 | 0.170 | 0.190 | — | — | — | — | — | — | — | — |
| Carbon emission | | kg CO_2_ | 7.335 | 7.716 | 9.245 | 7.186 | 12.493 | 9.656 | 14.481 | 2.381 | 9.488 | 10.668 | 10.206 | 10.717 | 11.705 | 12.704 | 8.629 |

**Table 5.1.7.** Consumption and carbon emissions of cast-in-place concrete stair works

Unit: 10m^2^

| Project | | | Straight | Arced | Spiral |
| --- | --- | --- | --- | --- | --- |
| Name | | Unit | Consumption / emissions | | |
| Material | Water | m^3^ | 0.722 | 0.696 | 0.591 |
|  | Electricity | kW·h | 1.560 | 1.580 | 1.590 |
| Carbon emission | | kg CO_2_ | 1.882 | 1.874 | 1.786 |

**Table 5.1.8.** Consumption and carbon emissions of cast-in-place concrete after-cast strip works

Unit: 10m^3^

| Project | | | Beam | Plate | Wall | Raft foundation |
| --- | --- | --- | --- | --- | --- | --- |
| Name | | Unit | Consumption / emissions | | | |
| Material | Water | m^3^ | 3.591 | 6.078 | 2.521 | 3.261 |
|  | Electricity | kW·h | 3.690 | 3.580 | 3.630 | 2.350 |
| Carbon emission | | kg CO_2_ | 6.164 | 8.341 | 5.144 | 4.812 |

**Table 5.1.9.** Consumption and carbon emissions of cast-in-place concrete other works

| Project | | | Water dispersion | Steps | Stadium stand | Trench | Handrail & pressure top | Small component |
| --- | --- | --- | --- | --- | --- | --- | --- | --- |
|  |  |  | Unit: 10m^2^ | | Unit: 10m^3^ | | | |
| Name | | Unit | Consumption / emissions | | | | | |
| Material | Water | m^3^ | 3.435 | 0.139 | 7.878 | 2.174 | 8.322 | 8.718 |
|  | Electricity | kW·h | 0.030 | 0.462 | 5.310 | 6.000 | — | — |
| Machine | Concrete smoothing machine | Team | 0.040 | — | — | — | — | — |
| Carbon emission | | kg CO_2_ | 3.876 | 0.489 | 11.337 | 6.688 | 7.573 | 7.933 |

In conclusion, we can obtain the carbon reduction and percentages of application technology of high-strength & high-performance concrete (**Table 5.1.10**).

**Table 5.1.10.** Carbon reduction and percentages of application technology of high-strength & high-performance concrete

Unit: 10m^3^

| Project | | | | C30 | High-strength & high-performance concrete | | | | |
| --- | --- | --- | --- | --- | --- | --- | --- | --- | --- |
|  |  |  |  |  | C60 | C65 | C70 | C75 | C80 |
| Name | | | Unit | Relative carbon emission | | | | | |
| Foundation | Cushion layer | | kg CO_2_ | - | -0.500 | -0.538 | -0.571 | -0.600 | -0.625 |
|  | Strip foundation | Rubble concrete | kg CO_2_ | - | -2.704 | -2.910 | -3.088 | -3.245 | -3.380 |
|  |  | Concrete | kg CO_2_ | - | -1.201 | -1.292 | -1.371 | -1.441 | -1.501 |
|  | Independent foundation | Rubble concrete | kg CO_2_ | - | -1.366 | -1.470 | -1.560 | -1.639 | -1.708 |
|  |  | Concrete | kg CO_2_ | - | -1.274 | -1.370 | -1.454 | -1.528 | -1.592 |
|  | Cup foundation | | kg CO_2_ | - | -1.419 | -1.526 | -1.620 | -1.702 | -1.773 |
|  | Raft foundation | Beamed type | kg CO_2_ | - | -1.453 | -1.563 | -1.659 | -1.743 | -1.816 |
|  |  | Beamless type | kg CO_2_ | - | -1.834 | -1.973 | -2.094 | -2.201 | -2.293 |
|  | Pile cap | | kg CO_2_ | - | -1.871 | -2.013 | -2.136 | -2.245 | -2.338 |
|  | Equipment Foundation (unit: m^3^) | ≤5 | kg CO_2_ | - | -1.419 | -1.526 | -1.620 | -1.702 | -1.773 |
|  |  | ≤20 | kg CO_2_ | - | -1.414 | -1.521 | -1.615 | -1.697 | -1.768 |
| Column | Rectangular column | | kg CO_2_ | - | -0.410 | -0.441 | -0.468 | -0.491 | -0.512 |
|  | Structural column | | kg CO_2_ | - | -1.809 | -1.946 | -2.065 | -2.170 | -2.261 |
|  | Special-shaped column | | kg CO_2_ | - | -2.274 | -2.447 | -2.597 | -2.729 | -2.843 |
|  | Circular column | | kg CO_2_ | - | -2.274 | -2.447 | -2.597 | -2.729 | -2.843 |
|  | Skew column | | kg CO_2_ | - | -2.207 | -2.374 | -2.520 | -2.648 | -2.758 |
|  | Steel pipe column | | kg CO_2_ | - | -2.286 | -2.460 | -2.611 | -2.743 | -2.858 |
|  | Steel reinforced concrete column | | kg CO_2_ | - | -1.460 | -1.571 | -1.667 | -1.752 | -1.825 |
| Beam | Foundation connecting beam | | kg CO_2_ | - | -1.828 | -1.967 | -2.088 | -2.194 | -2.285 |
|  | Rectangular beam | | kg CO_2_ | - | -2.844 | -3.060 | -3.247 | -3.412 | -3.554 |
|  | Special-shaped beam | | kg CO_2_ | - | -2.874 | -3.092 | -3.282 | -3.449 | -3.593 |
|  | Cantilever beam | | kg CO_2_ | - | -2.932 | -3.155 | -3.348 | -3.518 | -3.665 |
|  | Girth | | kg CO_2_ | - | -3.199 | -3.442 | -3.653 | -3.838 | -3.998 |
|  | Lintel | | kg CO_2_ | - | -3.363 | -3.618 | -3.840 | -4.035 | -4.203 |
|  | Arc & arched beam | | kg CO_2_ | - | -4.232 | -4.553 | -4.832 | -5.078 | -5.289 |
|  | Skew beam | | kg CO_2_ | - | -3.186 | -3.428 | -3.638 | -3.823 | -3.983 |
|  | Steel reinforced concrete beam | | kg CO_2_ | - | -3.195 | -3.437 | -3.648 | -3.833 | -3.993 |
| Wall | Straight wall | Rubble concrete | kg CO_2_ | - | -3.006 | -3.234 | -3.432 | -3.607 | -3.757 |
|  |  | Concrete | kg CO_2_ | - | -1.408 | -1.514 | -1.607 | -1.689 | -1.759 |
|  | Curved concrete wall | | kg CO_2_ | - | -1.704 | -1.833 | -1.945 | -2.044 | -2.129 |
|  | Short-leg shear wall | | kg CO_2_ | - | -1.743 | -1.875 | -1.990 | -2.091 | -2.178 |
|  | Retaining wall (gravity type) | Rubble concrete | kg CO_2_ | - | -1.731 | -1.863 | -1.977 | -2.077 | -2.164 |
|  |  | Rubble concrete | kg CO_2_ | - | -1.444 | -1.554 | -1.649 | -1.733 | -1.805 |
|  | Retaining wall (thin-walled) | Rubble concrete | kg CO_2_ | - | -1.751 | -1.884 | -1.999 | -2.101 | -2.188 |
|  | Elevator shaft wall straight wall | | kg CO_2_ | - | -2.688 | -2.892 | -3.070 | -3.226 | -3.360 |
|  | Climbing mold concrete wall | | kg CO_2_ | - | -1.912 | -2.057 | -2.183 | -2.294 | -2.389 |
|  | Laminated slab concrete composite wall | | kg CO_2_ | - | -2.383 | -2.564 | -2.721 | -2.860 | -2.979 |
|  | Large mold built-in insulation panel wall | | kg CO_2_ | - | -1.751 | -1.884 | -1.999 | -2.101 | -2.188 |
| Plate | Beamed plate | | kg CO_2_ | - | -1.751 | -1.884 | -1.999 | -2.101 | -2.188 |
|  | Beamless plate | | kg CO_2_ | - | -3.668 | -3.946 | -4.188 | -4.401 | -4.584 |
|  | Plain slab | | kg CO_2_ | - | -3.858 | -4.151 | -4.406 | -4.630 | -4.823 |
|  | Arch plate | | kg CO_2_ | - | -4.623 | -4.974 | -5.279 | -5.547 | -5.778 |
|  | Shell plate | | kg CO_2_ | - | -3.593 | -3.866 | -4.103 | -4.312 | -4.491 |
|  | Composite hollow plate | | kg CO_2_ | - | -6.247 | -6.721 | -7.134 | -7.496 | -7.808 |
|  | Inclined plate & slope roof plate | | kg CO_2_ | - | -4.828 | -5.195 | -5.514 | -5.794 | -6.035 |
|  | Rail plate | | kg CO_2_ | - | -7.241 | -7.791 | -8.269 | -8.689 | -9.051 |
|  | Bay window plate | | kg CO_2_ | - | -1.191 | -1.281 | -1.360 | -1.429 | -1.488 |
|  | Hanging plate | | kg CO_2_ | - | -4.744 | -5.105 | -5.418 | -5.693 | -5.930 |
|  | Pick eaves & gutter | | kg CO_2_ | - | -5.334 | -5.739 | -6.091 | -6.401 | -6.668 |
|  | Awning plate | | kg CO_2_ | - | -5.103 | -5.491 | -5.828 | -6.124 | -6.379 |
|  | Cantilever plate | | kg CO_2_ | - | -5.359 | -5.766 | -6.119 | -6.430 | -6.698 |
|  | Balcony plate | | kg CO_2_ | - | -5.853 | -6.297 | -6.684 | -7.023 | -7.316 |
|  | Patch seams between precast panels | | kg CO_2_ | - | -6.352 | -6.835 | -7.254 | -7.622 | -7.940 |
| Stair  (unit: 10m^2^) | Straight | | kg CO_2_ | - | -4.315 | -4.642 | -4.927 | -5.177 | -5.393 |
|  | Arc | | kg CO_2_ | - | -0.941 | -1.013 | -1.075 | -1.129 | -1.176 |
|  | Spiral | | kg CO_2_ | - | -0.937 | -1.008 | -1.070 | -1.124 | -1.171 |
| After-cast strip | Beam | | kg CO_2_ | - | -0.893 | -0.961 | -1.020 | -1.072 | -1.116 |
|  | Plate | | kg CO_2_ | - | -3.082 | -3.316 | -3.520 | -3.698 | -3.853 |
|  | Wall | | kg CO_2_ | - | -4.171 | -4.487 | -4.763 | -5.005 | -5.213 |
|  | Raft foundation | | kg CO_2_ | - | -2.572 | -2.767 | -2.937 | -3.086 | -3.215 |
| Others | Water dispersion (unit: 10m^2^) | | kg CO_2_ | - | -2.406 | -2.589 | -2.748 | -2.887 | -3.008 |
|  | steps | | kg CO_2_ | - | -1.938 | -2.085 | -2.213 | -2.326 | -2.423 |
|  | Stadium stand | | kg CO_2_ | - | -0.245 | -0.263 | -0.279 | -0.293 | -0.306 |
|  | Trench | | kg CO_2_ | - | -5.669 | -6.099 | -6.473 | -6.802 | -7.086 |
|  | Handrail & pressure top | | kg CO_2_ | - | -3.344 | -3.598 | -3.819 | -4.013 | -4.180 |
|  | Small component | | kg CO_2_ | - | -3.787 | -4.074 | -4.324 | -4.544 | -4.733 |
| Relative percentage of carbon emissions | | | % | - | -50.000 | -53.846 | -57.143 | -60.000 | -62.500 |

1. **Application technology of high strength bar**

High-strength steel bars refer to ordinary hot-rolled ribbed bars (HRB400 and HBR500) with yield strengths of 400 MPa and 500 MPa as specified in the national standard *Steel for the reinforcement of concrete-Part 2: Hot rolled ribbed bars* (GB/T 1499.2-2018). Through the comparison and calculation of the application of high-strength bars in various structures, the average amount of bars can be reduced by about 12%-18%, which has a good material saving effect. CRB600H high strength cold rolled ribbed steel bar is a new type of cold rolled ribbed bar developed in China in recent years. CRB600H high-strength rebar and HRB400 rebar price is comparable, but after application can further save steel up to 10%. Affected by the reduction of the amount, the consumption of materials and machinery required is also reduced, leading to the reduction of carbon emissions during the construction process. (**Table 5.2.1**)

**Table 5.2.1.** Relations between bar consumption and their strength grades

|  | Ordinary bar | HRB400/HRB500 | CRB600H |
| --- | --- | --- | --- |
| Relative consumption | 0 | -0.15 | -0.235 |

Affected by the reduction of the amount of bar, the consumption of materials and machinery required is also reduced, leading to the reduction of carbon emissions during the construction process. Refer to the *BC&DEC*, it is known that the engineering consumption required for the construction of bars of different diameters (**Table 5.2.2**) :

**Table 5.2.2.** Consumption and carbon emissions of cast-in-place ribbed bar works

Unit: t

| Project | | | | Diameters | | | |
| --- | --- | --- | --- | --- | --- | --- | --- |
|  |  |  |  | ≤ 10 | ≤ 18 | ≤ 25 | ≤ 40 |
| Name | | Unit | | Consumption / emissions | | | |
| Material | Water | | m^3^ | — | 0.144 | 0.093 | — |
| Machine | Steel bar straightener 40 mm | | Team | 0.270 | — | — | — |
|  | Steel bar cutting machine 40 mm | | Team | 0.110 | 0.100 | 0.180 | 0.130 |
|  | Steel bar bending machine 40 mm | | Team | 0.310 | 0.230 | 0.180 | 0.130 |
|  | Dc arc welding machine 32 kV·A | | Team | — | 0.450 | 0.400 | — |
|  | Butt welding machine 75 kV·A | | Team | — | 0.110 | 0.060 | — |
|  | Welding rod drying box 45×35×45 (cm^3^) | | Team | — | 0.045 | 0.040 | — |
| Carbon emission | | | kg CO_2_ | 8.409 | 48.798 | 41.776 | 4.582 |

In conclusion, we can obtain the carbon reduction and percentages of application technology of high strength bar (**Table 5.2.3**).

**Table 3.** Carbon reduction and percentages of application technology of high strength bar

Unit: kgCO_2_/t

| Project | High strength bar | |
| --- | --- | --- |
|  | HRB400/HRB500 | CRB600H |
| Name | Carbon reduction | |
| Diameter ≤ 10 mm | -1.261 | -1.976 |
| Diameter ≤ 18 mm | -7.320 | -11.468 |
| Diameter ≤ 25 mm | -6.266 | -9.817 |
| Diameter ≤ 40 mm | -0.687 | -1.077 |
| Percentage (%) | -15.000 | -23.500 |

1. **Application technology of high strength steel**

The selection of high-strength steel (ReL≥390Mpa) can reduce the amount of steel and processing, save resources and reduce costs. The national standard *High strength low alloy structural steels*（GB/T 1591-2018） stipulates eight grades, of which Q390, Q420, Q460, Q500, Q550, Q620, Q690 belong to the high strength steel range, *Steel plate for building structure*（GB/T 19879-2015） has Q390GJ, Q420GJ, Q460GJ three grades belong to the high strength steel range. Referring to the calculation of the amount of concrete in high-strength and high-performance concrete, similarly, the relationship between the amount of high strength steel and the strength grade can be obtained (**Table 5.3.1**):

**Table 5.3.1**. Relations between steel consumption and their strength grades

|  | Ordinary steel | | Higu strength steel | | | | | | |
| --- | --- | --- | --- | --- | --- | --- | --- | --- | --- |
| Stength grade | Q235GJ | Q355GJ | Q390GJ | Q420GJ | Q460GJ | Q500GJ | Q550GJ | Q620GJ | Q690GJ |
|  | Q235 | Q355 | Q390 | Q420 | Q460 | Q500 | Q550 | Q620 | Q690 |
| Relative consumption | 0.511 | 0.000 | -0.090 | -0.155 | -0.228 | -0.290 | -0.355 | -0.427 | -0.486 |

Affected by the reduction in the amount of steel, the consumption of materials and machinery required is also reduced, leading to a reduction in carbon emissions during the construction process. Refer to the *BC&DEC*, it is known that the material and machinery consumption required for the construction of each steel (**Table 5.3.2 - 5.3.3**) :

**Table 5.3.2.** Consumption and carbon emissions of steel structure of factory & warehouse building

Unit: t

| Project | | | Steel roof truss (Steel bracket) (unit: t) | | | | | Steel truss (unit: t) | | | | | | Steel platform | Steel stair | |
| --- | --- | --- | --- | --- | --- | --- | --- | --- | --- | --- | --- | --- | --- | --- | --- | --- |
|  |  |  | ≤1.5 | ≤ 3 | ≤ 8 | ≤ 15 | ≤ 25 | ≤ 1.5 | ≤ 3 | ≤ 8 | ≤ 15 | ≤ 25 | ≤ 40 |  | Step stairs | Climbing stairs |
| Name | | Unit | Consumption / emissions | | | | | | | | | | | | | |
| Material | CO_2_ gas | m^3^ | 0.715 | 0.715 | 0.858 | 0.858 | 1.210 | 2.002 | 1.650 | 1.210 | 1.210 | 2.002 | 2.002 | — | — | — |
| Machine | Truck crane 20 t | Team | 0.299 | 0.234 | 0.195 | — | — | 0.312 | 0.234 | 0.273 | — | — | — | 0.247 | 0.195 | 0.208 |
|  | Truck crane 40 t | Team | — | — | — | 0.195 | — | — | — | — | 0.234 | — | — | — | — | — |
|  | Ac arc welding machine 32 kV·A | Team | 0.110 | 0.110 | 0.132 | 0.165 | 0.264 | 0.308 | 0.253 | 0.198 | 0.198 | 0.308 | 0.308 | 0.308 | 0.308 | 0.308 |
|  | CO_2_ gas shielded welding machine 500 A | Team | 0.110 | 0.110 | 0.132 | 0.132 | 0.198 | 0.308 | 0.253 | 0.198 | 0.198 | 0.308 | 0.308 | — | — | — |
|  | Crawler crane 50 t | Team | — | — | — | — | 0.325 | — | — | — | — | 0.390 | 0.468 | — | — | — |
| Carbon emission | | kg CO_2_ | 49.968 | 42.239 | 40.483 | 49.088 | 75.096 | 77.456 | 60.985 | 58.256 | 60.943 | 93.517 | 104.150 | 52.712 | 46.528 | 48.074 |

Continued table

| Project | | | Steel column (unit: t) | | | | Steel beam (unit: t) | | | | Steel crane beams (unit: t) | | | | Steel supports &  other components | Steel wall frame | Piecemeal steel member |
| --- | --- | --- | --- | --- | --- | --- | --- | --- | --- | --- | --- | --- | --- | --- | --- | --- | --- |
|  |  |  | ≤ 3 | ≤ 8 | ≤ 15 | ≤ 25 | ≤ 1.5 | ≤ 3 | ≤ 8 | ≤ 15 | ≤ 3 | ≤ 8 | ≤ 15 | ≤ 25 |  |  |  |
| Name | | Unit | Consumption / emissions | | | | | | | | | | | | | | |
| Material | CO_2_ gas | m^3^ | 0.715 | 0.715 | 0.715 | 0.858 | 2.002 | 1.210 | 1.078 | 1.210 | 1.430 | 1.275 | 1.430 | 1.716 | — | — | — |
| Machine | Truck crane 20 t | Team | 0.156 | 0.130 | — | — | 0.234 | 0.156 | 0.221 | — | 0.234 | 0.195 | — | — | 0.234 | 0.221 | 0.273 |
|  | Truck crane 40 t | Team | — | — | 0.195 | — | — | — | — | 0.195 | — | — | 0.156 | — | — | — | — |
|  | Ac arc welding machine 32 kV·A | Team | 0.110 | 0.092 | 0.110 | 0.132 | 0.308 | 0.198 | 0.165 | 0.198 | 0.220 | 0.150 | 0.220 | 0.164 | 0.308 | 0.198 | 0.308 |
|  | CO_2_ gas shielded welding machine 500 A | Team | 0.110 | 0.092 | 0.110 | 0.132 | 0.308 | 0.198 | 0.165 | 0.198 | 0.220 | 0.150 | 0.220 | 0.164 | — | — | — |
|  | Crawler crane 50 t | Team | — | — | — | 0.260 | — | — | — | — | — | — | — | 0.260 | — | — | — |
| Carbon emission | | kg CO_2_ | 32.963 | 27.741 | 43.705 | 52.737 | 68.181 | 44.343 | 47.910 | 55.084 | 56.651 | 43.426 | 52.258 | 58.194 | 51.166 | 41.284 | 55.803 |

**Table 5.3.3.** Consumption and carbon emissions of high-rise steel structure

Unit: t

| Project | | | Steel column (unit: t) | | | | Steel beam (unit: t) | | | | Steel bracing (unit: t) | | | | Steel bracing (unit: t) |
| --- | --- | --- | --- | --- | --- | --- | --- | --- | --- | --- | --- | --- | --- | --- | --- |
|  |  |  | ≤3 | ≤5 | ≤10 | ≤15 | ≤0.5 | ≤1.5 | ≤3 | ≤5 | ≤1.5 | ≤3 | ≤5 | ≤8 |  |
| Name | | Unit | Consumption / emissions | | | | | | | | | | | | |
| Material | CO_2_ gas | m^3^ | 2.420 | 2.090 | 1.870 | 2.200 | 1.870 | 1.870 | 1.760 | 1.650 | 2.750 | 2.420 | 1.980 | 2.750 | 2.090 |
| Machine | Truck crane 40 t | Team | 0.026 | 0.026 | 0.026 | 0.026 | 0.026 | 0.026 | 0.026 | 0.026 | 0.026 | 0.026 | 0.026 | 0.026 | 0.026 |
|  | Ac arc welding machine 32 kV·A | Team | 0.187 | 0.180 | 0.170 | 0.190 | 0.280 | 0.250 | 0.220 | 0.220 | 0.250 | 0.220 | 0.200 | 0.220 | 0.275 |
|  | CO_2_ gas shielded welding machine 500 A | Team | 0.209 | 0.190 | 0.170 | 0.200 | 0.170 | 0.170 | 0.150 | 0.140 | 0.240 | 0.210 | 0.180 | 0.240 | 0.275 |
| Carbon emission | | kg CO_2_ | 31.446 | 29.464 | 27.426 | 30.862 | 35.761 | 33.488 | 30.149 | 29.509 | 38.182 | 33.989 | 30.339 | 35.909 | 40.283 |

In conclusion, we can obtain the carbon reduction and percentages of application technology of high strength steel (**Table 5.2.3**).

**Table 4.** Carbon reduction and percentages of application technology of high strength steel

Unit: kgCO_2_/t

| Project | | | Application technology of high strength steel | | | | | | |
| --- | --- | --- | --- | --- | --- | --- | --- | --- | --- |
|  |  |  | Q390GJ | Q420GJ | Q460GJ | Q500GJ | Q550GJ | Q620GJ | Q690GJ |
| Name | | | Carbon reduction | | | | | | |
| Factory  steel structure | Steel roof truss  (Steel bracket) (unit: t) | ≤ 1.5 | -4.484 | -7.733 | -11.406 | -14.491 | -17.716 | 49.541 | -24.260 |
|  |  | ≤ 3 | -3.791 | -6.537 | -9.641 | -12.249 | -14.976 | 41.812 | -20.507 |
|  |  | ≤ 8 | -3.633 | -6.265 | -9.241 | -11.740 | -14.353 | 40.056 | -19.655 |
|  |  | ≤ 15 | -4.405 | -7.597 | -11.205 | -14.236 | -17.404 | 48.661 | -23.833 |
|  |  | ≤ 25 | -6.739 | -11.622 | -17.141 | -21.778 | -26.625 | 74.669 | -36.460 |
|  | Steel truss (unit: t) | ≤ 1.5 | -6.951 | -11.987 | -17.680 | -22.462 | -27.462 | 77.029 | -37.606 |
|  |  | ≤ 3 | -5.473 | -9.438 | -13.920 | -17.686 | -21.622 | 60.558 | -29.609 |
|  |  | ≤ 8 | -5.228 | -9.016 | -13.298 | -16.894 | -20.655 | 57.829 | -28.284 |
|  |  | ≤ 15 | -5.469 | -9.432 | -13.911 | -17.673 | -21.607 | 60.516 | -29.588 |
|  |  | ≤ 25 | -8.392 | -14.473 | -21.346 | -27.120 | -33.156 | 93.090 | -45.403 |
|  |  | ≤ 40 | -9.346 | -16.118 | -23.773 | -30.204 | -36.926 | 103.723 | -50.566 |
|  | Steel column (unit: t) | ≤ 3 | -2.958 | -5.101 | -7.524 | -9.559 | -11.687 | 32.536 | -16.004 |
|  |  | ≤ 8 | -2.489 | -4.293 | -6.332 | -8.045 | -9.836 | 27.314 | -13.469 |
|  |  | ≤ 15 | -3.922 | -6.764 | -9.976 | -12.674 | -15.496 | 43.278 | -21.219 |
|  |  | ≤ 25 | -4.733 | -8.162 | -12.038 | -15.294 | -18.698 | 52.310 | -25.604 |
|  | Steel beam (unit: t) | ≤ 1.5 | -6.119 | -10.552 | -15.563 | -19.772 | -24.174 | 67.754 | -33.103 |
|  |  | ≤ 3 | -3.979 | -6.863 | -10.122 | -12.859 | -15.722 | 43.916 | -21.529 |
|  |  | ≤ 8 | -4.299 | -7.415 | -10.936 | -13.894 | -16.986 | 47.483 | -23.261 |
|  |  | ≤ 15 | -4.943 | -8.525 | -12.573 | -15.974 | -19.530 | 54.657 | -26.744 |
|  | Steel crane beams (unit: t) | ≤ 3 | -5.084 | -8.767 | -12.931 | -16.429 | -20.086 | 56.224 | -27.505 |
|  |  | ≤ 8 | -3.897 | -6.721 | -9.912 | -12.594 | -15.397 | 42.999 | -21.084 |
|  |  | ≤ 15 | -4.690 | -8.087 | -11.928 | -15.155 | -18.528 | 51.831 | -25.372 |
|  |  | ≤ 25 | -5.222 | -9.006 | -13.283 | -16.876 | -20.633 | 57.767 | -28.254 |
|  | Steel platform | | -4.730 | -8.158 | -12.032 | -15.286 | -18.689 | 52.285 | -25.592 |
|  | Steel stair | Step stairs | -4.175 | -7.201 | -10.620 | -13.493 | -16.497 | 46.101 | -22.590 |
|  |  | Climbing stairs | -4.314 | -7.440 | -10.973 | -13.941 | -17.045 | 47.647 | -23.340 |
|  | Steel supports & other components | | -4.592 | -7.918 | -11.679 | -14.838 | -18.141 | 50.739 | -24.842 |
|  | Steel wall frame | | -3.705 | -6.389 | -9.423 | -11.972 | -14.637 | 40.857 | -20.044 |
|  | Piecemeal steel member | | -5.008 | -8.636 | -12.738 | -16.183 | -19.785 | 55.376 | -27.093 |
| High-rise  steel structure | Steel column (unit: t) | ≤ 3 | -2.822 | -4.867 | -7.178 | -9.119 | -11.149 | 31.019 | -15.267 |
|  |  | ≤ 5 | -2.644 | -4.560 | -6.725 | -8.545 | -10.446 | 29.037 | -14.305 |
|  |  | ≤ 10 | -2.461 | -4.244 | -6.260 | -7.954 | -9.724 | 26.999 | -13.316 |
|  |  | ≤ 15 | -2.770 | -4.776 | -7.045 | -8.950 | -10.942 | 30.435 | -14.984 |
|  | Steel beam (unit: t) | ≤ 1.5 | -3.209 | -5.534 | -8.163 | -10.371 | -12.679 | 35.334 | -17.362 |
|  |  | ≤ 3 | -3.005 | -5.183 | -7.644 | -9.712 | -11.873 | 33.061 | -16.259 |
|  |  | ≤ 8 | -2.706 | -4.666 | -6.882 | -8.743 | -10.689 | 29.722 | -14.638 |
|  |  | ≤ 15 | -2.648 | -4.567 | -6.736 | -8.558 | -10.462 | 29.082 | -14.327 |
|  | Steel bracing (unit: t) | ≤ 1.5 | -3.426 | -5.909 | -8.715 | -11.073 | -13.537 | 37.755 | -18.538 |
|  |  | ≤ 3 | -3.050 | -5.260 | -7.758 | -9.857 | -12.051 | 33.562 | -16.502 |
|  |  | ≤ 5 | -2.723 | -4.695 | -6.925 | -8.798 | -10.757 | 29.912 | -14.730 |
|  |  | ≤ 8 | -3.222 | -5.557 | -8.197 | -10.414 | -12.732 | 35.482 | -17.434 |
|  | Step steel stairs | | -3.615 | -6.234 | -9.195 | -11.682 | -14.282 | 39.856 | -19.558 |
| Percentage (%) | | | -8.974 | -15.476 | -22.826 | -29.000 | -35.455 | -42.742 | -48.551 |

**Appendix 6. Carbon emissions of simplified construction processes technologies**

1. **High-strength bar straight thread connection technology**

Straight thread mechanical connection is the main way of high-strength steel bar connection, the process is to use the connection sleeve first with a straight thread head with a pipe wrench or installation wrench to apply a certain tightening torque to screw together, so that it meets the requirements of the minimum tightening torque value of the specifications of the joint specified in the industry standard, and the steel wire head in the central position of the sleeve to each other. In the construction process, the welding process of traditional electroslag pressure welding technology and gas pressure welding is subtracted by this technology, which reduces the consumption of electricity and reduces the carbon emission in the construction process. See *BC&DEC*, the engineering consumption required for traditional electroslag pressure welding technology, gas pressure welding technology and straight thread connection technology (**Table 6.1.1**) :

**Table 6.1.1.** Consumption and carbon emissions of traditional electroslag pressure welding technology, gas pressure welding technology and straight thread connection technology

Unit: 10

| Project | | | Electroslag pressure welding (diameter: mm) | | Gas pressure welding (diameter: mm) | | Straight thread connection (diameter: mm) | | | | |
| --- | --- | --- | --- | --- | --- | --- | --- | --- | --- | --- | --- |
|  |  |  | ≤ 18 | ≤ 32 | ≤ 25 | ≤ 40 | ≤ 16 | ≤ 20 | ≤ 25 | ≤ 32 | ≤ 40 |
| Name | | Unit | Consumption / emissions | | | | | | | | |
| Material | C_2_H_2_ gas | m^3^ | — | — | 1.620 | 1.700 | — | — | — | — | — |
|  | Electricity | kW·h | — | — | 0.700 | 0.750 | — | — | — | — | — |
| Machine | Electroslag welding machine 1000 A | Team | 0.060 | 0.070 | — | — | — | — | — | — | — |
|  | Voltage welding machine | Team | — | — | 0.250 | 0.270 | — | — | — | — | — |
|  | Bolt sleeve wire machine | Team | — | — | — | — | 0.140 | 0.160 | 0.180 | 0.210 | 0.250 |
| Carbon emission | | kg CO_2_ | 6.924 | 8.078 | 15.423 | 16.456 | 2.748 | 3.140 | 3.533 | 4.121 | 4.906 |
| Relative carbon emission | | kg CO_2_ | 0.000 | 1.154 | 8.499 | 9.532 | -4.177 | -3.784 | -3.392 | -2.803 | -2.018 |
| Relative percentage of carbon emission | | % | 0.000 | 16.623 | 122.429 | 137.309 | -60.170 | -54.509 | -48.989 | -40.482 | -29.145 |

1. **Mechanical anchorage technology of bar**

Mechanical anchorage technology of bar is the anchoring device formed by connecting the anchoring plate with the end of the steel bar through the thread, thus reducing the anchoring length of the steel bar and simplifying the construction process of reinforcement anchoring. The anchor plate reinforcement mainly replaces the traditional curved reinforcement and straight reinforcement, and is used for the shear reinforcement of beam column joints, simple beam supports, beams and plates of frame structure. This technology can reduce the bending, welding, drying and other processes in the traditional bending bar anchorage. See *BC&DEC,* the engineering consumption required for traditional flexural anchorage and mechanical anchorage (**Table 6.2.1**) :

**Table 6.2.1.** Consumption and carbon emissions of traditional flexural anchorage technology and mechanical anchoring technology

Unit: 10

| Project | | | Traditional flexural anchorage (diameter: mm) | | | | Mechanical anchoring (diameter: mm) | | | |
| --- | --- | --- | --- | --- | --- | --- | --- | --- | --- | --- |
|  |  |  | ≤ 10 | ≤ 18 | ≤ 25 | ≤ 40 | ≤ 10 | ≤ 18 | ≤ 25 | ≤ 40 |
| Name | | Unit | Consumption / emissions | | | | | | | |
| Material | Water | m^3^ | — | 0.144 | 0.093 | — | — | 0.144 | 0.093 | — |
| Machine | Steel bar straightener 40 mm | Team | 0.270 | — | — | — | 0.270 | — | — | — |
|  | Steel bar cutting machine 40 mm | Team | 0.110 | 0.100 | 0.180 | 0.130 | 0.110 | 0.100 | 0.180 | 0.130 |
|  | Steel bar bending machine 40 mm | Team | 0.310 | 0.230 | 0.180 | 0.130 | — | — | — | — |
|  | Dc arc welding machine 32 kV·A | Team | — | 0.450 | 0.400 | — | — | — | — | — |
|  | Butt welding machine 75 kV·A | Team | — | 0.110 | 0.060 | — | — | — | — | — |
|  | Welding rod drying box 45×35×45 (cm^3^) | Team | — | 0.045 | 0.040 | — | — | — | — | — |
| Carbon emission | | kg CO_2_ | 8.409 | 48.798 | 41.776 | 4.582 | 5.294 | 2.651 | 4.620 | 3.276 |
| Relative carbon emission | | kg CO_2_ | 0.000 | 0.000 | 0.000 | 0.000 | -3.115 | -46.147 | -37.156 | -1.306 |
| Relative percentage of carbon emission | | % | 0.000 | 0.000 | 0.000 | 0.000 | -37.044 | -94.567 | -88.941 | -28.503 |

1. **Long auger hole pressure pile technology**

In the long auger hole pressure pile technology, the long auger drill is used to drill to the design elevation, the concrete pump is used to press the superfluid fine stone concrete from the bottom of the drill, and the drill bit is lifted until the pile is formed. After the concrete is poured to the design elevation, the reinforcement cage is inserted into the concrete pile once to the design elevation by means of the reinforcement cage weight or a special vibration device, and formed reinforced concrete cast-in-place pile. Compared with ordinary underwater pile construction technology, the construction of long auger hole pressure pile does not need mud wall protection, no mud skin, no sediment, no mud pollution, and the construction speed is fast, the cost is low. Currently, the common construction technology of underwater grouting piles is Slurry-supported bored piles, which is the construction technology of rotary drilling machine to form holes and pour concrete. The construction technology of long auger hole pressure pile technology is the auger hole drilling and concrete pouring. Refer to *BC&DEC*, it can be seen that the consumption of materials and machinery required for the construction of cast-in-place pile (**Table 6.3.1**) :

**Table 6.3.1.** Consumption and carbon emissions of traditional rotary drill hole mud wall protection drill pile technology and long auger hole pressure pile technology

Unit: 10 m^3^

| Project | | | | Traditional rotary drill hole mud wall protection drill pile technology (diameter: mm) | | | | Long auger hole pressure pile technology (diameter: mm) | | |
| --- | --- | --- | --- | --- | --- | --- | --- | --- | --- | --- |
|  |  |  |  | ≤ 800 | ≤ 1000 | ≤ 1200 | ≤ 1500 | ≤ 400 | ≤ 600 | ≤ 800 |
| Name | | | Unit | Consumption / emissions | | | | | | |
| Phase | Material | Water | m^3^ | 27.600 | 26.560 | 26.560 | 22.100 | — | — | — |
| Hole drilling | Machine | Rotary drill 800 mm | Team | 1.937 | — | — | — | — | — | — |
|  |  | Rotary drill 1000 mm | Team | — | 1.498 | — | — | — | — | — |
|  |  | Rotary drill 1500 mm | Team | — | — | 1.059 | 0.840 | — | — | — |
|  |  | Auger 400 mm | Team | — | — | — | — | 0.880 | — | — |
|  |  | Auger 600 mm | Team | — | — | — | — | — | 0.670 | — |
|  |  | Auger 800 mm | Team | — | — | — | — | — | — | 0.580 |
|  |  | Mud pump 100 mm | Team | 1.937 | 1.498 | 1.059 | 0.840 | — | — | 0.580 |
|  |  | Ac arc welding machine 32 kV·A | Team | 0.160 | 0.140 | 0.140 | 0.120 | 0.192 | 0.168 | 0.168 |
| Concrete pouring | Machine | Crawler crane 25t | Team | 0.171 | 0.171 | 0.171 | 0.171 | 0.171 | 0.171 | 0.171 |
| Carbon emission | | | kg CO_2_ | 629.835 | 522.752 | 407.931 | 329.238 | 119.427 | 127.647 | 231.843 |
| Relative carbon emission | | | kg CO_2_ | 0.000 | -107.083 | -221.904 | -300.597 | -510.408 | -502.188 | -397.992 |
| Relative percentage of carbon emission | | | % | 0.000 | -17.002 | -35.232 | -47.726 | -81.038 | -79.733 | -63.190 |

1. **Combined aluminum alloy formwork technology**

Aluminum alloy formwork is a kind of light weight, high strength, high processing accuracy, a single piece of large format, less joint, convenient construction, and has a wide range of applications, can be poured at the same time, high surface quality of molded concrete, less construction waste advantages. Aluminum alloy formwork meets the requirements of building industrialization, environmental protection and energy saving. Compared with the traditional combination formwork, the combined aluminum alloy formwork can be reused to greatly reduce construction waste, thereby reducing the need for waste transportation at the construction site and reducing the carbon emissions at the construction site. Refer to *BC&DEC*, it is known that the formwork, wood support and other materials consumed by every 100 m^2^ formwork construction are the construction waste that needs to be transported outside after construction,and the carbon emissions of waste transportation (**Table 6.4.1**):

**Table 6.4.1.** Consumption and carbon emissions of traditional combination formwork and combined aluminum alloy formwork

Unit: 100m^2^

| Project | | | Traditional combination formwork | | | | | | | Combined aluminum alloy formwork technology | | | | | | |
| --- | --- | --- | --- | --- | --- | --- | --- | --- | --- | --- | --- | --- | --- | --- | --- | --- |
|  |  |  | Rectangular column | Special-  shaped column | Rectangular beam | Special-  shaped beam | Straight wall | Plate | Integral stairs (unit: 10 m^2^) | Rectangular column | Special-  shaped column | Rectangular beam | Special-  shaped beam | Straight wall | Plate | Integral stairs (unit: 10 m^2^) |
| Name | | Unit | Consumption / emissions | | | | | | | | | | | | | |
| Material | Composite formwork | m^2^ | 24.675 | 30.629 | 24.675 | 24.675 | 24.675 | 24.675 | 52.719 | — | — | — | — | — | — | — |
|  | Steel support and accessories | kg | 12.327 | 25.690 | 103.441 | 103.282 | 18.743 | 56.353 | 65.360 | — | — | — | — | — | — | — |
|  | Wood | m^3^ | 0.342 | 0.377 | — | — | 0.790 | 0.259 | 0.946 | — | — | — | — | — | — | — |
|  | Galvanized channel steel | kg | 29.035 | — | — | — | — | — | — | — | — | — | — | — | — | — |
|  | Round nails | kg | 0.982 | 1.220 | 1.224 | 29.570 | 18.557 | 1.149 | 2.408 | — | — | — | — | — | — | — |
|  | Tension bolt | kg | 21.638 | 31.040 | 10.750 | 15.276 | 13.546 | — | — | 19.340 | 22.713 | — | — | — | — | — |
|  | Plastic hard pipe *φ*20 | m | 132.500 | 117.766 | 14.193 | 149.235 | 63.443 | — | — | — | — | — | — | — | — | — |
|  | Steel parts (comprehensive) | kg | — | — | — | — | 2.529 | — | — | — | — | — | — | — | — | — |
|  | Pin pieces | Set | — | — | — | — | — | — | — | 80.400 | 84.422 | 80.520 | 84.546 | 80.400 | 74.400 | 26.141 |
|  | Steel back 60×40×2.5 | kg | — | — | — | — | — | — | — | 6.824 | 7.712 | 1.418 | 1.601 | 6.824 | — | 34.215 |
|  | Diagonal supporting rod *φ*48×3.5 | Set | — | — | — | — | — | — | — | 0.480 | 0.423 | — | — | 0.449 | — | 0.238 |
|  | Aluminum formwork | kg | — | — | — | — | — | — | — | 28.987 | 32.757 | 29.111 | 32.890 | 28.987 | 27.071 | 9.391 |
|  | Sporadic fixtures | kg | — | — | — | — | — | — | — | 7.344 | 7.712 | 0.918 | 1.037 | 8.976 | — | — |
|  | Pull pieces | kg | — | — | — | — | — | — | — | — | — | 21.318 | 24.084 | 26.436 | — | — |
|  | Vertical support member *φ*48×3.5 | Set | — | — | — | — | — | — | — | — | — | 0.969 | 1.013 | — | 0.857 | — |
| Construction waste volume | | m^3^ | 1.084 | 1.262 | 0.602 | 0.655 | 1.635 | 0.923 | 2.474 | 0.038 | 0.035 | 0.068 | 0.071 | 0.033 | 0.060 | 0.022 |
| Carbon emission | | kg CO_2_ | 22.099 | 25.728 | 12.268 | 13.357 | 33.327 | 18.824 | 50.437 | 0.775 | 0.708 | 1.391 | 1.455 | 0.666 | 1.226 | 0.456 |
| Relative carbon emission | | kg CO_2_ | 0.000 | 0.000 | 0.000 | 0.000 | 0.000 | 0.000 | 0.000 | -21.324 | -25.020 | -10.877 | -11.902 | -32.661 | -17.597 | -49.981 |
| Relative percentage of carbon emission | | % | 0.000 | 0.000 | 0.000 | 0.000 | 0.000 | 0.000 | 0.000 | -96.493 | -97.248 | -88.662 | -89.107 | -98.002 | -93.482 | -99.096 |

1. **Combined ribbed plastic formwork technology**

The plastic formwork has the characteristics of smooth surface, easy demoulding, light weight, good corrosion resistance, many turnover times, and recyclable, which is conducive to environmental protection and meets the national requirements of energy conservation and environmental protection. The Combined ribbed plastic formwork wall column adopts steel back, and the horizontal formwork adopts the support system composed of independent support, early removal head or steel beam, which can realize early removal of formwork, convenient construction, safe and reliable. This technology is similar to the construction technology of combined aluminum alloy formwork technology, and the consumption and carbon emissions can be referred to **Table 6.4.1**.

1. **One time forming technology of concrete floor**

The one time forming technology of concrete floor is to use *φ*150mm steel pipe to roll and lift the pulp after the concrete pouring is completed, scrape the bar to adjust the flatness, or use laser automatic leveling and mechanical pulping methods, spread wear-resistant mixture (fine steel sand, steel fiber, etc.) before the concrete floor is set, use the polishing machine to smooth, and finally carry out the modification process. Compared with the traditional construction technology, the one time forming technology saves the leveling layer, and has a remarkable effect on shortening the construction period, saving materials and reducing carbon emission in the construction process. Refer to *BC&DEC* to see the consumption of materials and machinery required for the construction of every 100 m^2^ of leveling layer (**Table 6.6.1**) :

**Table 6.6.1**. Consumption and carbon emissions of leveling layer construction in traditional ground construction

Unit: 100 m^2^

| Project | | | Leveling layer construction in traditional ground construction | | | | |
| --- | --- | --- | --- | --- | --- | --- | --- |
|  |  |  | Mortar screed | | | Fine stone concrete screed | |
|  |  |  | On a concrete or hard base | On the filling material | Each increase or decrease 1 mm | 30 mm | Each increase or decrease 1 mm |
|  |  |  | 20 mm | |  |  |  |
| Name | | Unit | Consumption / emissions | | | | |
| Material | Water | m^3^ | 0.910 | 1.038 | — | 0.400 | — |
| Machine | Dry mortar tank mixer | Team | 0.204 | 0.255 | 0.010 | — | — |
| Carbon emission | | kg CO_2_ | 5.394 | 6.652 | 0.224 | 0.364 | 0.000 |

1. **No plastering technology of building walls**

No plastering technology of building walls refers to the use of new formwork system, new wall materials or the use of prefabricated walls, so that the surface quality reach the quality level of plaster-free or direct decoration. Compared with traditional wall construction, this technology saves wall plastering, and has significant effect on shortening construction period, saving materials and reducing carbon emission during construction. Refer to *BC&DEC* to see the consumption of materials and machinery required for every 100 m^2^ wall plastering construction (**Table 6.7.1**) :

**Table 6.7.1.** Consumption and carbon emissions of plaster in traditional wall construction

Unit: 100 m^2^

| Project | | | Common plaster | | | | | External wall decoration plaster | |
| --- | --- | --- | --- | --- | --- | --- | --- | --- | --- |
|  |  |  | Interior wall | Outer wall | Rubble wall | Light wall | Each increase or decrease 1 mm | Granitic plaster | Artificial stone |
|  |  |  | （14+6）mm | | （24+6）mm | （15+5）mm |  |  |  |
| Name | | Unit | Consumption / emissions | | | | | | |
| Material | Water | m^3^ | 0.980 | 0.980 | 1.203 | 0.935 | 0.029 | 3.159 | 0.968 |
| Machine | Dry mortar tank mixer | Team | 0.232 | 0.232 | 0.321 | 0.214 | 0.012 | 0.129 | 0.129 |
|  | Mortar mixer 200 L | Team | 0.000 | 0.000 | 0.000 | 0.000 | 0.000 | 0.172 | 0.172 |
| Carbon emission | | kg CO_2_ | 6.084 | 6.084 | 8.279 | 5.640 | 0.295 | 6.924 | 4.930 |

**Appendix 7. Carbon emissions of renewable resources & waste reduction technologies**

1. **Water collection & comprehensive utilization technology**

The water collection & comprehensive utilization technology at the construction site includes excavation construction precipitation recycling technology, rainwater recycling technology, on-site production and domestic wastewater recycling technology. This technology can significantly reduce the need for traditional municipal water supplies, thereby reducing carbon emissions. Refer to Appendix 1, it can be seen that the carbon emission coefficient of municipal water supply is 0.91 kg CO_2_ / m^3^.

1. **Construction waste reduction & resource utilization technology**

The construction waste reduction technology refers to the adoption of new green construction technology, fine construction and standardized construction measures in the construction process to reduce construction waste emissions; The resource utilization technology of construction waste refers to the nearby disposal of construction waste, recycling direct utilization or processing and reuse. It can be found that the technology has two main advantages of reducing the consumption of construction raw materials and reducing the transportation of construction waste. The reduction of carbon emissions caused by reducing the consumption of building raw materials mainly occurs in the production plant, not the construction site. Therefore, the main impact on the carbon emissions of the construction site is to reduce the carbon emissions generated during the transportation of construction waste. See *BC&DEC*, it can be seen that the mechanical consumption required for the external transportation of 10m^3^ construction waste (**Table 7.2.1**) :

**Table 7.2.1.** Consumption and carbon emissions of transportation of construction waste

Unit: 10m^3^

| Project | | | Transport waste from floors (vertical) | | Transport waste outward (horizontal) | |
| --- | --- | --- | --- | --- | --- | --- |
|  |  |  | Vertical transport distance within 18 m | Each additional 1 m | Horizontal transport distance within 5 km | Each additional 1 km |
| Name | | Unit | Consumption / emissions | | | |
| Machine | Wheeled loader 0.5 m^3^ | Team | — | — | 0.800 | — |
|  | Dump truck 8 t | Team | — | — | 0.444 | 0.036 |
|  | Single cage construction elevator 1 t 75 m | Team | 0.960 | 0.053 | — | — |
| Carbon emission | | kg CO_2_ | 31.892 | 1.761 | 171.955 | 4.562 |

1. **Solar energy & Air energy utilization technology**

This technology mainly includes construction site solar photovoltaic lighting technology, solar hot water application technology and air energy hot water technology etc.. These technologies can significantly increase the proportion of clean energy used in the construction process and reduce the dependence on municipal electricity. Refer to Appendix 1, it can be seen that the carbon emission coefficient of municipal electricity supply is 0.785 kg CO_2_ / kW·h.

**Appendix 8. Carbon emissions of prefabrication technologies**

1. **Application technology of steel bar welding mesh**

The steel bar welding mesh is a steel mesh with the same or different diameters of longitudinal and transverse steel bars arranged vertically at a certain distance, and all the crossing points are welded together by resistance spot welding. The use of welding mesh can significantly improve the quality of steel bar engineering, reduce the working hours of on-site steel bar installation, shorten the construction period, save steel properly, and have better comprehensive economic benefits. Compared with traditional steel bar construction, the construction process of steel bar welding network reduces the procedures of steel bar straightening, cutting, bending and welding on the construction site. See *BC&DEC* to see the consumption of materials and machinery required for each t of construction using the application technology of steel bar welding mesh (**Table 8.1.1**) :

**Table 8.1.1.** Consumption and carbon emissions of traditional steel welding technology and application technology of steel bar welding mesh

Unit: t

| Project | | | Traditional steel welding technology (diameter: mm) | | | | Application technology of steel bar welding mesh (diameter: mm) | | | |
| --- | --- | --- | --- | --- | --- | --- | --- | --- | --- | --- |
|  |  |  | ≤ 10 | ≤ 18 | ≤ 25 | ≤ 40 | ≤ 10 | ≤ 18 | ≤ 25 | ≤ 40 |
| Name | | Unit | Consumption / emissions | | | | | | | |
| Material | Water | m^3^ | — | 0.144 | 0.093 | — | — | 0.144 | 0.093 | — |
| Machine | Steel bar straightener 40 mm | Team | 0.270 | — | — | — | — | — | — | — |
|  | Steel bar cutting machine 40 mm | Team | 0.110 | 0.100 | 0.180 | 0.130 | — | — | — | — |
|  | Steel bar bending machine 40 mm | Team | 0.310 | 0.230 | 0.180 | 0.130 | — | — | — | — |
|  | Dc arc welding machine 32 kV·A | Team | — | 0.450 | 0.400 | — | — | — | — | — |
|  | Butt welding machine 75 kV·A | Team | — | 0.110 | 0.060 | — | — | — | — | — |
|  | Welding rod drying box 45×35×45 (cm^3^) | Team | — | 0.045 | 0.040 | — | — | — | — | — |
| Carbon emission | | kg CO_2_ | 8.409 | 48.798 | 41.776 | 4.582 | 0.000 | 0.131 | 0.085 | 0.000 |
| Relative carbon emission | | kg CO_2_ | 0.000 | 0.000 | 0.000 | 0.000 | -8.409 | -48.667 | -41.691 | -4.582 |
| Relative percentage of carbon emission | | % | 0.000% | 0.000% | 0.000% | 0.000% | -100.000% | -99.732% | -99.797% | -100.000% |

1. **Processing & distribution technology of molding steel bar products**

The processing & distribution technology of molding steel bar products refers to a kind of steel bar processing method with the characteristics of modern construction industrialization, which is carried out by specialized steel bar processing institutions with information production management system for large-scale industrial and professional production and commercial distribution of steel bars. This technology is similar to the application technology of steel bar welding mesh, which also reduces the processes of steel bar straightening, cutting, bending and welding on the construction site, and the consumption and carbon emissions can be referred to **Table 8.1.1**.

1. **Precast segmental box girder formwork technology**

Precast segmental box girder refers to the whole span beam is divided into different segments, prefabricated in the prefabrication plant, transported to the beam frame site, and assembled into holes by special segment assembly bridge erecting machine, hole by hole construction is completed. Compared with the traditional field formwork technology, the precast segmental box girder formwork can be prefabricated in the factory in advance, reducing the amount of formwork on the construction site, shortening the construction period, and having better comprehensive economic benefits. Moreover, most of the precast segmental box girder formwork uses fixed steel formwork, which can be recycled and reused, greatly reducing construction waste, thus reducing the demand for waste transportation at the construction site, and reducing the carbon emissions at the construction site. Refer to the *Municipal Engineering Consumption Quota* (ZYA-1-31-2015) Volume 3 *Bridge and Culvert Engineering*, it is known that the formwork, wood support and other materials consumed by every 100 m^2^ formwork construction, namely the construction waste that needs to be transported outside after construction (**Table 8.3.1**).

**Table 8.3.1.** Consumption and carbon emissions of traditional field formwork technology and precast segmental box girder formwork technology

Unit: 100 m^2^

| Project | | | Traditional field formwork technology | Precast segmental box girder formwork technology |
| --- | --- | --- | --- | --- |
| Name | | Unit | Consumption / emissions | |
| Material | Wood | m^3^ | 0.720 | 0.050 |
|  | Steel bar (within *φ*10) | kg | — | 3.000 |
|  | Shaped steel formwork | kg | — | 354.090 |
|  | Welded steel pipe (General) | kg | — | 3.000 |
|  | Steel parts (comprehensive) | kg | — | 53.000 |
|  | Semi-hard plastic pipe *φ*32 | m | — | 10.300 |
|  | composite formwork | m^2^ | 24.680 | — |
|  | Steel support and accessories | kg | 47.540 | — |
|  | Round nails | kg | 1.790 | — |
| Construction waste volume | | m^3^ | 1.244 | 0.066 |
| Carbon emission | | kg CO_2_ | 25.365 | 1.340 |
| Relative carbon emission | | kg CO_2_ | 0.000 | -24.025 |
| Relative percentage of carbon emission | | % | 0.000 | -94.717 |

1. **Prefabricated concrete shear wall structure technology**

Prefabricated concrete shear wall structure refers to the whole concrete shear wall structure which adopts prefabricated wall members in whole or in part, and forms the whole concrete shear wall structure by pouring concrete and cement-based grouting material through reliable connection method. The technology mainly includes lifting, correcting and fixing of components, straightening of joint steel bars, laying of slurry materials, caulking and other processes, which reduces the steel bar engineering, support mold engineering and concrete pouring engineering of traditional cast-in-place concrete shear walls, reduces the energy consumption of construction sites, and also reduces the carbon emissions of transportation of construction waste such as formwork. Refer to the *BC&DEC* to see the material and machinery consumption required for the construction of 10 m^3^ shear wall (**Table 8.4.1**) :

**Table 8.4.1.** Consumption and carbon emissions of traditional cast-in-place concrete shear wall and prefabricated concrete shear wall structure technology

Unit: 10m^3^

| Project | | | Traditional cast-in-place concrete shear wall | Prefabricated concrete shear wall structure technology |
| --- | --- | --- | --- | --- |
| Name | | Unit | Consumption / emissions | |
| Material | composite formwork | m^2^ | 23.441 | — |
|  | Wood | m^3^ | 0.751 | — |
|  | Steel support and accessories | kg | 17.806 | — |
|  | Round nails | kg | 17.629 | — |
|  | Steel parts (comprehensive) | kg | 4.594 | — |
|  | Plastic hard pipe *φ*20 | m | 60.271 | — |
|  | Tension bolt | kg | 12.869 | — |
|  | Pad iron | kg | — | 12.491 |
|  | PE rod | m | — | 40.751 |
|  | Crosser | m^3^ | — | 0.012 |
|  | Diagonal supporting rod *φ*48×3.5 | Set | — | 0.487 |
|  | Embedded iron parts | kg | — | 9.307 |
|  | Positioning steel plate | kg | — | 4.550 |
|  | Water (bar engineering) | m^3^ | 0.170 | — |
|  | Water (concrete engineering) | m^3^ | 0.587 | — |
|  | Electricity | kW·h | 3.730 | — |
| Machine | Steel bar cutting machine 40 mm | Team | 0.164 | — |
|  | Steel bar bending machine 40 mm | Team | 0.329 | — |
|  | Dc arc welding machine 32 kV·A | Team | 0.731 | — |
|  | Butt welding machine 75 kV·A | Team | 0.110 | — |
|  | Welding rod drying box 45×35×45 (cm^3^) | Team | 0.073 | — |
|  | Dry mortar tank mixer | Team | — | 0.010 |
| Construction waste volume | | m^3^ | 1.554 | 0.050 |
| Carbon emission | | kg CO_2_ | 107.312 | 1.239 |
| Relative carbon emission | | kg CO_2_ | 0.000 | -106.073 |
| Relative percentage of carbon emission | | % | 0.000 | -98.845 |

1. **Prefabricated concrete frame structure technology**

Prefabricated concrete frame structure includes the assembled monolithic concrete frame structure and other prefabricated concrete frame structures. The prefabricated integral frame structure refers to the concrete structure in which all or part of the frame beams and columns are assembled by prefabricated components through reliable connection methods, and the components are connected into an integral whole by in-situ pouring concrete and cement-based grouting materials at the connection nodes. Other assembled frames mainly refer to all kinds of dry-connected frame structures, which are mainly used in conjunction with shear walls and seismic supports. This technology reduces the steel bar engineering, support form engineering and concrete pouring engineering of traditional cast-in-place concrete frame structure construction, reduces the energy consumption of the construction site, and also reduces the carbon emission of transportation of construction waste such as formwork. Refer to the *BC&DEC* to see the material and machinery consumption required for the construction of 10 m^3^ concrete frame structure (**Table 8.5.1**) :

**Table 8.5.1.** Consumption and carbon emissions of traditional cast-in-place concrete frame structure technology and prefabricated concrete frame structure technology

Unit: 10m^3^

| Project | | | Traditional cast-in-place concrete frame structure technology | | Prefabricated concrete frame structure technology | |
| --- | --- | --- | --- | --- | --- | --- |
|  |  |  | Column | Beam | Column | Beam |
| Name | | Unit | Consumption / emissions | | | |
| Material | Composite formwork | m^2^ | 23.840 | 22.514 | — | — |
|  | Steel support and accessories | kg | 11.910 | 94.381 | — | — |
|  | Wood | m^3^ | 0.330 | 0.356 | — | — |
|  | Galvanized channel steel | kg | 28.053 | — | — | — |
|  | Round nails | kg | 0.949 | 1.117 | — | — |
|  | Tension bolt | kg | 20.906 | 9.808 | — | — |
|  | Plastic hard pipe *φ*20 | m | 128.019 | 12.950 | — | — |
|  | Crosser | m^3^ | — | — | 0.010 | — |
|  | Diagonal supporting rod *φ*48×3.5 | Set | — | — | 0.340 | — |
|  | Embedded iron parts | kg | — | — | 13.050 | — |
|  | Pad iron | kg | — | — | 7.480 | 3.270 |
|  | Wood | m^3^ | — | — | — | 0.014 |
|  | Vertical support member *φ*48×3.5 | Set | — | — | — | 1.040 |
|  | Sporadic fixtures | kg | — | — | — | 9.360 |
|  | Water (bar engineering) | m^3^ | 0.161 | 0.173 | — | — |
|  | Water (concrete engineering) | m^3^ | 0.774 | 3.090 | — | — |
|  | Electricity | kW·h | 3.710 | 3.740 | — | — |
| Machine | Steel bar cutting machine 40 mm | Team | 0.156 | 0.167 | — | — |
|  | Steel bar bending machine 40 mm | Team | 0.312 | 0.334 | — | — |
|  | Dc arc welding machine 32 kV·A | Team | 0.692 | 0.743 | — | — |
|  | Butt welding machine 75 kV·A | Team | 0.104 | 0.111 | — | — |
|  | Welding rod drying box 45×35×45 (cm^3^) | Team | 0.069 | 0.074 | — | — |
|  | Dry mortar tank mixer | Team | — | — | 0.008 | — |
| Construction waste volume | | m^3^ | 1.047 | 1.012 | 0.037 | 0.093 |
| Carbon emission | | kg CO_2_ | 93.357 | 99.777 | 0.931 | 1.893 |
| Relative carbon emission | | kg CO_2_ | 0.000 | 0.000 | -92.426 | -97.884 |
| Relative percentage of carbon emission | | % | 0.000 | 0.000 | -99.003 | -98.103 |

1. **Concrete laminated floor technology**

Concrete laminated floor technology refers to the floor is divided into two parts along the thickness direction, the bottom is a prefabricated floor, the upper is a post-cast concrete laminated layer. The prefabricated bottom plate with the bottom reinforcement is a part of the floor, and during the construction phase, it acts as a formwork for the post-cast concrete laminated layer to bear the load and form an integral laminated concrete member with the post-cast concrete layer. Compared with the traditional cast-in-place floor construction, this technology reduces the steel bar engineering, support form engineering and concrete pouring engineering in the construction, reduces the energy consumption of the construction site, and also reduces the carbon emissions of transportation of construction waste such as formwork. Refer to the *BC&DEC* to see the material and machinery consumption required for the construction of 10 m^3^ concrete floor structure (**Table 8.6.1**) :

**Table 8.6.1.** Consumption and carbon emissions of traditional cast-in-place floor technology and concrete laminated floor technology

Unit: 10m^3^

| Project | | | Traditional cast-in-place floor technology | Concrete laminated floor technology |
| --- | --- | --- | --- | --- |
| Name | | Unit | Consumption / emissions | |
| Material | Composite formwork | m2 | 19.491 | — |
|  | Wood | m3 | 0.217 | — |
|  | Steel support and accessories | kg | 56.462 | 39.850 |
|  | Sporadic fixtures | kg | 9.901 | 37.310 |
|  | Round nails | kg | 0.908 | — |
|  | Plastic hard pipe *φ*20 | m | 12.738 | — |
|  | Tension bolt | kg | 1.626 | — |
|  | Pad iron | kg | — | 3.140 |
|  | Wood | m3 | — | 0.091 |
|  | Vertical support member *φ*48×3.5 | Set | — | 2.730 |
|  | Water (bar engineering) | m3 | 0.117 | — |
|  | Water (concrete engineering) | m3 | 2.595 | — |
|  | Electricity | kW·h | 3.790 | — |
| Machine | Steel bar cutting machine 40 mm | Team | 0.113 | — |
|  | Steel bar bending machine 40 mm | Team | 0.226 | — |
|  | Dc arc welding machine 32 kV·A | Team | 0.503 | — |
|  | Butt welding machine 75 kV·A | Team | 0.075 | — |
|  | Welding rod drying box 45×35×45 (cm^3^) | Team | 0.050 | — |
|  | Concrete smoothing machine | Team | 0.110 | — |
|  | Woodworking circular saw | Team | 0.029 | — |
|  | Ac arc welding machine 32 kV·A | Team | — | 0.581 |
| Construction waste volume | | m^3^ | 0.753 | 0.317 |
| Carbon emission | | kg CO_2_ | 69.395 | 48.989 |
| Relative carbon emission | | kg CO_2_ | 0.000 | -20.405 |
| Relative percentage of carbon emission | | % | 0.000 | -29.406 |

1. **Precast concrete wall hanging panel technology**

Precast concrete wall hanging panel is a non-load-bearing precast concrete wall panel which is installed on the main structure and plays the role of enclosure and decoration. Precast concrete wall hanging panels can be used in brick veneer, stone veneer, colored concrete veneer, clear concrete veneer, bare bone concrete veneer and concrete veneer with decorative patterns on the surface of the wall, which can make the building wall have a unique expression. The precast concrete wall hanging panel is produced in industrial mode in the factory. Compared with the construction of traditional cast-in-place wall hanging panel, this technology reduces the steel bar engineering, support form engineering and concrete pouring engineering in the construction, reduces the energy consumption of the construction site, and also reduces the carbon emissions of transportation of construction waste such as formwork. Refer to the *BC&DEC* to see the material and machinery consumption required for the construction of precast concrete wall hanging panels per 10 m^3^ (**Table 8.7.1**) :

**Table 8.7.1.** Consumption and carbon emissions of traditional cast-in-place concrete wall panels and precast concrete wall hanging panel technology

Unit: 10m^3^

| Project | | | Traditional cast-in-place concrete wall panels | Precast concrete wall hanging panel technology | |
| --- | --- | --- | --- | --- | --- |
|  |  |  |  | Wall thickness ≤ 200mm | Wall thickness ＞200mm |
| Name | | Unit | Consumption / emissions | | |
| Material | Composite formwork | m2 | 54.169 | — | — |
|  | Wood | m3 | 0.357 | — | — |
|  | Steel support and accessories | kg | 15.698 | — | — |
|  | Round nails | kg | 3.012 | — | — |
|  | Sporadic fixtures | kg | 5.687 | — | — |
|  | Tension bolt | kg | 9.726 | — | — |
|  | Plastic hard pipe *φ*20 | m | 73.157 | — | — |
|  | Pad iron | kg | — | 24.528 | 15.322 |
|  | PE rod | m | — | 55.840 | 40.615 |
|  | Crosser | m3 | — | 0.020 | 0.020 |
|  | Diagonal supporting rod *φ*48×3.5 | Set | — | 0.821 | 0.598 |
|  | Embedded iron parts | kg | — | 15.697 | 11.417 |
|  | Water (bar engineering) | m3 | 3.010 | — | — |
|  | Electricity | kW·h | 10.100 | — | — |
| Machine | Steel bar straightener 40 mm | Team | 0.221 | — | — |
|  | Steel bar cutting machine 40 mm | Team | 0.090 | — | — |
|  | Steel bar bending machine 40 mm | Team | 0.254 | — | — |
|  | Woodworking circular saw | Team | 0.276 | — | — |
|  | Dry mortar tank mixer | Team | — | 0.010 | 0.010 |
| Construction waste volume | | m3 | 1.764 | 0.084 | 0.068 |
| Carbon emission | | kg CO2 | 62.218 | 1.929 | 1.610 |
| Relative carbon emission | | kg CO2 | 0.000 | -60.289 | -60.608 |
| Relative percentage of carbon emission | | % | 0.000 | -96.900 | -97.412 |

1. **Sandwich insulation wall panel technology**

Sandwich insulation wall panel refers to the composite wall panel formed between the insulation material and two layers of concrete wall panel (inner wall and outer wall), which can achieve the purpose of enhancing the energy-saving performance of the external wall insulation, reducing the fire risk of the external wall, improving the insulation life of the wall panel and reducing the maintenance cost of the external wall. Compared with the construction of traditional large mold built-in insulation panel wall, this technology reduces the construction of steel bar engineering, support mold engineering and concrete pouring engineering, reduces the energy consumption of the construction site, and also reduces the carbon emissions of transportation of construction waste such as formwork. Refer to the *BC&DEC* to see the material and machinery consumption required for the construction of sandwich insulation wall panel per 10 m^3^ (**Table 8.5.1**) :

**Table 8.8.1.** Consumption and carbon emissions of traditional large mold built-in insulation panel wall and sandwich insulation wall panel technology

Unit: 10m^3^

| Project | | | | Traditiona large mold built-in insulation panel wall | Sandwich insulation wall panel technology | |
| --- | --- | --- | --- | --- | --- | --- |
|  |  |  |  |  | Wall thickness ≤ 300mm | Wall thickness ＞ 300mm |
| Name | | Unit | | Consumption / emissions | | |
| Material | Shaped steel formwork | | kg | 80.829 | — | — |
|  | Steel support and accessories | | kg | 2.469 | — | — |
|  | Wood | | m^3^ | 0.023 | — | — |
|  | Sporadic fixtures | | kg | 4.387 | — | — |
|  | Steel parts (comprehensive) | | kg | 7.846 | — | — |
|  | Wood support | | m^3^ | 0.010 | — | — |
|  | Round nails | | kg | 1.387 | — | — |
|  | Tension bolt | | kg | 5.706 | — | — |
|  | Plastic hard pipe *φ*20 | | m | 45.299 | — | — |
|  | Pad iron | | kg | — | 9.243 | 8.393 |
|  | PE rod | | m | — | 24.476 | 22.248 |
|  | Crosser | | m^3^ | — | 0.015 | 0.015 |
|  | Diagonal supporting rod *φ*48×3.5 | | Set | — | 0.360 | 0.327 |
|  | Embedded iron parts | | kg | — | 6.880 | 6.254 |
|  | Positioning steel plate | | kg | — | 3.734 | 3.394 |
|  | Water (bar engineering) | | m^3^ | 0.095 | — | — |
|  | Water (concrete engineering) | | m^3^ | 0.690 | — | — |
|  | Electricity | | kW·h | 3.660 | — | — |
| Machine | Steel bar cutting machine 40 mm | | Team | 0.092 | — | — |
|  | Steel bar bending machine 40 mm | | Team | 0.185 | — | — |
|  | Dc arc welding machine 32 kV·A | | Team | 0.410 | — | — |
|  | Butt welding machine 75 kV·A | | Team | 0.062 | — | — |
|  | Welding rod drying box 45×35×45 (cm^3^) | | Team | 0.041 | — | — |
|  | Woodworking circular saw | | Team | 0.009 | — | — |
|  | Dry mortar tank mixer | | Team | — | 0.010 | 0.010 |
| Construction waste volume | | | m3 | 0.064 | 0.045 | 0.042 |
| Carbon emission | | | kg CO2 | 45.492 | 1.136 | 1.089 |
| Relative carbon emission | | | kg CO2 | 0.000 | -44.356 | -44.403 |
| Relative percentage of carbon emission | | | % | 0.000 | -97.503 | -97.606 |

1. **Superimposed shear wall structure technology**

Superimposed shear wall structure refers to the composite shear wall structure formed by using two layers of prefabricated wall panels with lattice rebar (truss rebar). After being installed in place, concrete is poured in the middle of the two layers of slab, supplemented by necessary cast-in-place concrete shear walls, edge members and floor slabs. Compared with the traditional cast-in-place shear wall construction, this technology reduces the steel bar engineering, support framework engineering and concrete pouring engineering in the construction, reduces the energy consumption of the construction site, and also reduces the carbon emission of transportation of construction waste such as formwork. Refer to the *BC&DEC* to know the material and machinery consumption required for the construction of 10 m^3^ superimposed shear wall structure (**Table 8.9.1**) :

**Table 8.9.1.** Consumption and carbon emissions of traditional cast-in-place shear wall technology and superimposed shear wall structure technology

Unit: 10m^3^

| Project | | | Traditional cast-in-place shear wall technology | Superimposed shear wall structure technology |
| --- | --- | --- | --- | --- |
| Name | | Unit | Consumption / emissions | |
| Material | Composite formwork | m^2^ | 23.433 | — |
|  | Wood | m^3^ | 0.750 | — |
|  | Steel support and accessories | kg | 17.800 | — |
|  | Round nails | kg | 17.623 | — |
|  | Steel parts (comprehensive) | kg | 4.593 | — |
|  | Plastic hard pipe *φ*20 | m | 60.250 | — |
|  | Tension bolt | kg | 12.864 | — |
|  | Pad iron | kg | — | 16.360 |
|  | Crosser | m^3^ | — | 0..013 |
|  | Hexagonal bolts with nuts (comprehensive) | kg | — | 8.080 |
|  | Wood | m^3^ | — | 0.038 |
|  | Diagonal supporting rod *φ*48×3.5 | Set | — | 0.350 |
|  | Embedded iron parts | kg | — | 13.420 |
|  | Water (bar engineering) | m^3^ | 0.170 | — |
|  | Water (concrete engineering) | m^3^ | 0.587 | — |
|  | Electricity | kW·h | 3.730 | — |
| Machine | Steel bar cutting machine 40 mm | Team | 0.164 | — |
|  | Steel bar bending machine 40 mm | Team | 0.329 | — |
|  | Dc arc welding machine 32 kV·A | Team | 0.731 | — |
|  | Butt welding machine 75 kV·A | Team | 0.110 | — |
|  | Welding rod drying box 45×35×45 (cm^3^) | Team | 0.073 | — |
|  | Woodworking circular saw | Team | 0.027 | — |
| Construction waste volume | | m^3^ | 1.553 | 0.091 |
| Carbon emission | | kg CO_2_ | 107.802 | 1.852 |
| Relative carbon emission | | kg CO_2_ | 0.000 | -105.934 |
| Relative percentage of carbon emission | | % | 0.000 | -98.282 |

1. **Precast prestressed concrete member technology**

Precast prestressed concrete members refer to all kinds of horizontal and vertical members produced by factories and using pretensioned prestressed technology, which mainly include: precast prestressed concrete hollow slab, precast prestressed concrete beam and precast prestressed concrete wall panel. The advantage of precast prestressed concrete members is that there is generally no support in the construction stage and the construction process is simplified. Compared with the traditional construction of cast-in-place prestressed concrete components, this technology reduces the steel bar engineering, support form engineering and concrete pouring engineering in the construction, reduces the energy consumption of the construction site, and also reduces the carbon emissions of transportation of construction waste such as formwork. Refer to the *BC&DEC* for the material and mechanical consumption required for the construction of prestressed beams, plates and walls per 10 m^3^ (**Table 8.10.1**) :

**Table 8.10.1.** Consumption and carbon emissions of traditional prestressed concrete component site construction technology and precast prestressed concrete member technology

Unit: 10m^3^

| Project | | | Traditional prestressed concrete component site construction technology | | | | | | Precast prestressed concrete member technology | | |
| --- | --- | --- | --- | --- | --- | --- | --- | --- | --- | --- | --- |
|  |  |  | Cast-in-place  prestressed concrete hollow plate | Cast-in-place prestressed concrete beam | | | | Cast-in-place  prestressed concrete wal | Precast  prestressed concrete hollow plate | Precast  prestressed  beam | Precast  prestressed concrete wall panels |
|  |  |  |  | Rectangular beam | Special-  shaped beam | Curved beam | Arched beam |  |  |  |  |
| Name | | Unit | Consumption / emissions | | | | | | | | |
| Material | Composite formwork | m^2^ | 19.491 | 22.514 | 22.091 | 21.992 | 19.172 | 23.433 | — | — | — |
|  | Wood | m^3^ | 0.205 | 0.356 | 0.334 | 0.401 | 0.319 | 0.750 | — | — | — |
|  | Steel support and accessories | kg | 44.513 | 94.381 | 92.464 | 130.060 | 88.276 | 17.800 | 23.910 | 10.000 | — |
|  | Round nails | kg | 0.908 | 1.117 | 26.473 | 37.227 | 0.951 | 17.623 | — | — | — |
|  | Galvanized iron wire *φ*0.7 | kg | 0.142 | 0.164 | 0.161 | 0.160 | 0.140 | — | — | — | — |
|  | Galvanized iron wire *φ*4 | kg | — | — | — | 29.599 | — | — | — | — | — |
|  | Steel parts (comprehensive) | kg | — | — | — | — | — | 2.402 | — | — | — |
|  | Plastic hard pipe *φ*20 | m | — | 12.950 | 133.620 | 22.228 | 18.655 | 60.250 | — | — | — |
|  | Tension bolt | kg | — | 9.808 | 13.676 | 6.109 | 5.130 | 12.864 | — | — | — |
|  | Steel back 60×40×2.5 | kg | — | — | — | 26.186 | — | — | — | — | — |
|  | Pad iron | kg | — | — | — | — | — | — | 1.880 | 3.270 | 7.695 |
|  | Wood | m^3^ | — | — | — | — | — | — | 0.055 | 0.014 | — |
|  | Sporadic fixtures | kg | — | — | — | — | — | — | 22.380 | 9.360 | — |
|  | PE rod | m | — | — | — | — | — | — | — | — | 40.615 |
|  | Crosser | m^3^ | — | — | — | — | — | — | — | — | 0.010 |
|  | Embedded iron parts | kg | — | — | — | — | — | — | — | — | 5.710 |
|  | Positioning steel plate | kg | — | — | — | — | — | — | — | — | 3.640 |
|  | Vertical support member *φ*48×3.5 | Set | — | — | — | — | — | — | 1.640 | 1.040 | — |
|  | Diagonal supporting rod *φ*48×3.5 | Set | — | — | — | — | — | — | — | — | 0.289 |
|  | Water (bar engineering) | m^3^ | 0.734 | 1.068 | 1.149 | 1.197 | 0.844 | 0.748 | — | — | — |
|  | Water (concrete engineering) | m^3^ | 3.958 | 3.090 | 3.200 | 3.759 | 3.759 | 0.587 | — | — | — |
|  | Electricity | kW·h | 3.790 | 3.740 | 3.760 | 3.760 | 3.760 | 3.660 | — | — | — |
| Machine | Concrete smoothing machine | Team | 0.170 | 0.170 | 0.170 | 0.170 | 0.170 | 0.170 | — | — | — |
|  | Steel bar cutting machine 40 mm | Team | 0.102 | 0.149 | 0.160 | 0.166 | 0.117 | 0.104 | — | — | — |
|  | lectric single-barrel slow winch 50 kN | Team | 0.727 | 1.059 | 1.139 | 1.186 | 0.837 | 0.741 | — | — | — |
|  | Butt welding machine 75 kV·A | Team | 0.453 | 0.660 | 0.709 | 0.739 | 0.521 | 0.462 | — | — | — |
|  | Prestressed steel tensile machine 650 kN | Team | 0.715 | 1.040 | 1.119 | 1.165 | 0.822 | 0.728 | — | — | — |
|  | Woodworking circular saw | Team | 0.066 | 0.034 | 0.733 | 0.951 | 0.257 | 0.009 | — | — | — |
|  | Ac arc welding machine 32 kV·A | Team | — | — | — | — | — | — | 0.349 | — | — |
|  | Dry mortar tank mixer | Team | — | — | — | — | — | — | — | — | 0.009 |
| Construction waste volume | | m^3^ | 0.729 | 1.012 | 1.021 | 1.072 | 0.886 | 1.552 | 0.191 | 0.093 | 0.033 |
| Carbon emission | | kg CO_2_ | 101.251 | 140.005 | 161.764 | 172.322 | 119.196 | 115.199 | 30.331 | 1.893 | 0.880 |
| Relative carbon emission | | kg CO_2_ | 0.000 | 0.000 | 0.000 | 0.000 | 0.000 | 0.000 | -109.674 | Rectangular beam: -138.112  Special-shaped beam: -159.871  Curved beam: -170.429  Arched beam: -117.303 | -114.319 |
| Relative percentage of carbon emission | | % | 0.000 | 0.000 | 0.000 | 0.000 | 0.000 | 0.000 | -70.044 | Rectangular beam: -98.648  Rectangular beam: -98.830  Curved beam: -98.901  Arc beam: -98.412 | -99.236 |

1. **Factory production and processing technology of prefabricated components**

The factory production and processing technology of prefabricated components refers to the technology that adopts automatic assembly line, unit assembly line and long line bench production line to produce standard shaped prefabricated components and take into account special-shaped prefabricated components, and adopts fixed bench mold line to produce prefabricated components for housing construction, so as to meet the mass production and processing and centralized supply requirements of prefabricated components. Components are processed in the processing plant in advance, transported to the site and directly constructed. Compared with the traditional on-site prefabricated component construction, this technology can reduce the carbon emissions generated by the fabrication of prefabricated components on the construction site, so as to achieve carbon emission reduction in construction. Refer to the *BC&DEC* to see the material and machinery consumption required for the construction of 10 m^3^ of prefabricated components (**Table 8.11.1**) :

**Table 8.11.1.** Consumption and carbon emissions of traditional field prefabricated component construction technology

Unit: 10m^3^

| Project | | | Traditional field prefabricated component construction technology | | | | |
| --- | --- | --- | --- | --- | --- | --- | --- |
|  |  |  | Lintel | Trench cover | Overhead heat shield | Hollow lattice | Small component |
| Name | | Unit | Consumption / emissions | | | | |
| Material | Crosser | m^3^ | 0.016 | 0.075 | 0.112 | 0.584 | 1.468 |
|  | Round nails | kg | 8.917 | 1.085 | 3.008 | 30.368 | 24.293 |
|  | Galvanized iron wire *φ*0.7 | kg | 0.436 | 0.212 | 0.729 | 0.368 | 1.179 |
|  | Composite formwork | m^2^ | 30.729 | 16.330 | 17.463 | 19.740 | 51.947 |
|  | Water (bar engineering) | m^3^ | 0.053 | 0.076 | 0.076 | 0.076 | 0.076 |
|  | Water (concrete engineering) | m^3^ | 3.602 | 5.550 | 9.069 | 9.492 | 7.910 |
|  | Electricity | kW·h | 1.500 | 1.750 | 1.750 | 1.500 | 1.500 |
| Machine | Steel bar cutting machine 40 mm | Team | 0.057 | 0.082 | 0.082 | 0.082 | 0.082 |
|  | Steel bar bending machine 40 mm | Team | 0.091 | 0.131 | 0.131 | 0.131 | 0.131 |
|  | Dc arc welding machine 32 kV·A | Team | 0.228 | 0.328 | 0.328 | 0.328 | 0.328 |
|  | Butt welding machine 75 kV·A | Team | 0.034 | 0.049 | 0.049 | 0.049 | 0.049 |
|  | Welding rod drying box 45×35×45 (cm^3^) | Team | 0.023 | 0.033 | 0.033 | 0.033 | 0.033 |
|  | Woodworking circular saw | Team | 0.057 | 0.025 | 0.026 | 0.773 | 1.065 |
|  | Woodworking single side press planer 600 mm | Team | 0.057 | 0.025 | 0.026 | 0.773 | 1.065 |
| Construction waste volume | | m^3^ | 0.740 | 0.480 | 0.554 | 1.221 | 3.124 |
| Carbon emission | | kg CO2 | 44.411 | 49.678 | 54.444 | 99.055 | 148.481 |

1. **Steel structure residential application technology**

The steel structure residential uses steel structure as the main bearing structure system of the house. For low-density residential buildings, cold-formed thin-wall steel structure system should be adopted. For multi-high-rise residential structures, steel frame, frame support (wall panel), barrel structure, steel frame-steel composite system, etc., the floor structure should adopt steel truss floor or assembled monolithic floor, and the wall should be prefabricated light slab or light block. Compared with traditional cast-in-place concrete structure residential construction, this technology can reduce the construction of columns and beams in the concrete works, steel works and formwork, instead of the installation of steel columns and steel beams, so as to achieve carbon reduction in construction. Refer to the *BC&DEC* to know the consumption of material and machinery required for the construction of each t of steel (Table 8.12.1-8.12.2) :

**Table 8.12.1.** Consumption and carbon emissions of traditional cast-in-place concrete structure residential application technology and steel structure residential application technology (column)

| Project | | | Traditional cast-in-place concrete structure residential application technology | | | | Steel structure residential application technology | | | |
| --- | --- | --- | --- | --- | --- | --- | --- | --- | --- | --- |
|  |  |  | Rectangular column | Structural column | Special-shaped column | Circular column | ≤ 3 | ≤ 5 | ≤ 10 | ≤ 15 |
|  |  |  | Unit: 10 m^3^ | | | | Unit: t | | | |
| Name | | Unit | Consumption / emissions | | | | | | | |
| Material | Composite formwork | m^2^ | 23.840 | 24.650 | 30.690 | — | — | — | — | — |
|  | Steel support and accessories | kg | 11.910 | 45.440 | 25.741 | 6.627 | — | — | — | — |
|  | Wood | m^3^ | 0.330 | 0.386 | 0.378 | — | — | — | — | — |
|  | Galvanized channel steel | kg | 28.053 | — | — | — | — | — | — | — |
|  | Wood support | m^3^ | — | 0.182 | — | — | — | — | — | — |
|  | Sporadic fixtures | kg | — | — | — | — | — | — | — | — |
|  | Round nails | kg | 0.949 | 0.982 | 1.222 | 0.915 | — | — | — | — |
|  | Tension bolt | kg | 20.906 | — | 31.102 | — | — | — | — | — |
|  | Plastic hard pipe *φ*20 | m | 128.019 | — | 118.002 | — | — | — | — | — |
|  | Pull hoop connectors | 个 | — | — | — | 22.280 | — | — | — | — |
|  | Shaped composite formwork 15 mm | m^2^ | — | — | — | 22.320 | — | — | — | — |
|  | Flat steel (comprehensive) | kg | — | — | — | 45.136 | — | — | — | — |
|  | Metal structure iron | kg | — | — | — | — | 10.588 | 7.344 | 6.528 | 5.610 |
|  | CO_2_ gas | m^3^ | — | — | — | — | 2.420 | 2.090 | 1.870 | 2.200 |
|  | Steel wire rope | kg | — | — | — | — | 3.690 | 3.690 | 3.690 | 5.690 |
|  | Crosser | m^3^ | — | — | — | — | 0.011 | 0.011 | 0.011 | 0.011 |
|  | Water (bar engineering) | m^3^ | 0.161 | 0.161 | 0.052 | 0.168 | — | — | — | — |
|  | Water (concrete engineering) | m^3^ | 0.774 | 1.789 | 1.789 | 1.658 | — | — | — | — |
|  | Electricity | kW·h | 3.710 | 3.720 | 3.720 | 3.700 | — | — | — | — |
| Machine | Steel bar cutting machine 40 mm | Team | 0.156 | 0.156 | 0.051 | 0.162 | — | — | — | — |
|  | Steel bar bending machine 40 mm | Team | 0.312 | 0.312 | 0.102 | 0.325 | — | — | — | — |
|  | Dc arc welding machine 32 kV·A | Team | 0.692 | 0.692 | 0.226 | 0.722 | — | — | — | — |
|  | Butt welding machine 75 kV·A | Team | 0.104 | 0.104 | 0.034 | 0.108 | — | — | — | — |
|  | Welding rod drying box 45×35×45 (cm^3^) | Team | 0.069 | 0.069 | 0.023 | 0.072 | — | — | — | — |
|  | Woodworking circular saw | Team | 0.053 | 0.055 | 0.055 | 0.041 | — | — | — | — |
|  | Truck crane 40 t | Team | — | — | — | — | 0.026 | 0.026 | 0.026 | 0.026 |
|  | Ac arc welding machine 32 kV·A | Team | — | — | — | — | 0.187 | 0.180 | 0.170 | 0.190 |
|  | CO_2_ gas shielded welding machine 500 A | Team | — | — | — | — | 0.209 | 0.190 | 0.170 | 0.200 |
| Construction waste volume | | m^3^ | 1.047 | 1.322 | 1.265 | 0.444 | 0.016 | 0.016 | 0.015 | 0.015 |
| Carbon emission | | kg CO_2_ | 94.358 | 100.921 | 53.649 | 85.550 | 32.015 | 30.022 | 27.981 | 31.414 |

**Table 8.12.12.** Consumption and carbon emissions of traditional cast-in-place concrete structure residential application technology and steel structure residential application technology (beam)

| Project | | | Traditional cast-in-place concrete structure residential application technology | | | | | | | Steel structure residential application technology | | | |
| --- | --- | --- | --- | --- | --- | --- | --- | --- | --- | --- | --- | --- | --- |
|  |  |  | Rectangular beam | Special-shaped beam | Girth | Lintel | Arched beam | Curved beam | Skew beam | ≤ 0.5 | ≤ 1.5 | ≤ 3 | ≤ 5 |
|  |  |  | Unit: 10 m^3^ | | | | | | | Unit: t | | | |
| Name | | Unit | Consumption / emissions | | | | | | | | | | |
| Material | Composite formwork | m^2^ | 22.514 | 22.091 | 20.242 | 33.435 | 19.172 | 21.992 | 22.514 | — | — | — | — |
|  | Steel support and accessories | kg | 94.381 | 92.464 | 4.858 | 67.249 | 88.276 | 130.060 | 63.394 | — | — | — | — |
|  | Wood | m^3^ | 0.356 | 0.334 | 0.510 | 0.814 | 0.319 | 0.401 | 0.408 | — | — | — | — |
|  | Round nails | kg | 1.117 | 26.473 | 1.298 | 2.070 | 0.951 | 37.227 | 1.038 | — | — | — | — |
|  | Plastic hard pipe *φ*20 | m | 12.950 | 133.620 | — | — | 18.655 | 22.228 | 12.950 | — | — | — | — |
|  | Tension bolt | kg | 9.808 | 13.676 | — | — | 5.130 | 6.109 | 3.776 | — | — | — | — |
|  | Steel back 60×40×2.5 | kg | — | — | — | — | — | 26.186 | — | — | — | — | — |
|  | Metal structure iron | kg | — | — | — | — | — | — | — | 7.344 | 6.936 | 6.528 | 5.712 |
|  | CO_2_ gas | m^3^ | — | — | — | — | — | — | — | 1.870 | 1.870 | 1.760 | 1.650 |
|  | Steel wire rope | kg | — | — | — | — | — | — | — | 3.280 | 3.280 | 3.280 | 3.280 |
|  | Crosser | m^3^ | — | — | — | — | — | — | — | 0.012 | 0.012 | 0.012 | 0.012 |
|  | Water (bar engineering) | m^3^ | 0.173 | 0.186 | 0.088 | 0.109 | 0.137 | 0.194 | 0.122 | — | — | — | — |
|  | Water (concrete engineering) | m^3^ | 3.090 | 3.200 | 4.640 | 6.065 | 3.759 | 3.759 | 3.795 | — | — | — | — |
|  | Electricity | kW·h | 3.740 | 3.760 | 3.188 | 3.750 | 3.760 | 3.760 | 3.740 | — | — | — | — |
| Machine | Steel bar cutting machine 40 mm | Team | 0.167 | 0.180 | 0.085 | 0.105 | 0.132 | 0.188 | 0.118 | — | — | — | — |
|  | Steel bar bending machine 40 mm | Team | 0.334 | 0.360 | 0.171 | 0.211 | 0.264 | 0.375 | 0.237 | — | — | — | — |
|  | Dc arc welding machine 32 kV·A | Team | 0.743 | 0.799 | 0.380 | 0.469 | 0.587 | 0.834 | 0.526 | — | — | — | — |
|  | Butt welding machine 75 kV·A | Team | 0.111 | 0.120 | 0.057 | 0.070 | 0.088 | 0.125 | 0.079 | — | — | — | — |
|  | Welding rod drying box 45×35×45 (cm^3^) | Team | 0.074 | 0.080 | 0.038 | 0.047 | 0.059 | 0.083 | 0.053 | — | — | — | — |
|  | Woodworking circular saw | Team | 0.034 | 0.733 | 0.007 | 0.237 | 0.257 | 0.951 | 0.034 | — | — | — | — |
|  | Truck crane 40 t | Team | 0.000 | — | — | — | — | — | 0.000 | 0.026 | 0.026 | 0.026 | 0.026 |
|  | Ac arc welding machine 32 kV·A | Team | 0.000 | — | — | — | — | — | 0.000 | 0.280 | 0.250 | 0.220 | 0.220 |
|  | CO_2_ gas shielded welding machine 500 A | Team | 0.000 | — | — | — | — | — | 0.000 | 0.170 | 0.170 | 0.150 | 0.140 |
| Construction waste volume | | m^3^ | 1.012 | 1.021 | 1.138 | 1.852 | 0.886 | 1.072 | 1.073 | 0.017 | 0.017 | 0.017 | 0.017 |
| Carbon emission | | kg CO_2_ | 100.413 | 119.422 | 67.551 | 96.990 | 87.267 | 128.467 | 80.816 | 36.346 | 34.071 | 30.731 | 30.088 |

1. [] Data source: *Building Carbon Emission Calculation Standar*d (GB/T 51366-2019), Ministry of Housing and Urban-Rural Development, PRC. [↑](#footnote-ref-1)
2. [] Data source: *Rules for the Compilation of Expenses of Construction Machinery for Construction Projects*, Ministry of Housing and Urban-Rural Development, PRC. [↑](#footnote-ref-2)
3. [] Data source: *National Unified Construction Project Foundation Quota (GJD-101-95),* Ministry of Housing and Urban-Rural Development, PRC. [↑](#footnote-ref-3)
4. [] Data source: *Building Construction and Decoration Engineering Consumption (TY 01-31-2021),* Ministry of Housing and Urban-Rural Development, PRC. [↑](#footnote-ref-4)
